# Gli1 regulates fibro/adipogenic progenitor function through modulation of Ido1 in muscle regeneration

**DOI:** 10.7150/ijbs.116134

**Published:** 2025-09-29

**Authors:** Lili Han, Fengmin Zhang, Jujin Zhang, Xiaonan Li, Kunpeng Wang, Biao Liu, Jiawen Song, Xinyan Liu, Yun Qian, Kai Li, Yingnan Lei, Claudia Spits, Yi Chang, Chengle Zhuang, Zhen Yu, Yun Zhao, Jiayin Peng

**Affiliations:** 1 Key Laboratory of Multi-Cell Systems, Shanghai Institute of Biochemistry and Cell Biology, Center for Excellence in Molecular Cell Science, Chinese Academy of Sciences, University of Chinese Academy of Sciences, Shanghai 200031, PR China; 2 Department of Gastrointestinal Surgery, Shanghai Tenth People's Hospital, School of Medicine, Tongji University, Shanghai 200072, PR China; 3 Key Laboratory of Systems Health Science of Zhejiang Province, School of Life Science, Hangzhou Institute for Advanced Study, University of Chinese Academy of Sciences, Hangzhou 310024, PR China; 4 Research Group Genetics, Reproduction and Development, Faculty of Medicine and Pharmacy, Vrije Universiteit Brussel, 1090, Brussels, Belgium; 5 Department of Medical Aesthetic, Yangpu Hospital, School of Medicine, Tongji University, Shanghai 200090, PR China; 6 Department of Gastrointestinal Surgery, Renji Hospital, Shanghai Jiao Tong University School of Medicine, Shanghai 200127, PR China; 7 School of Life Science and Technology, ShanghaiTech University, 100 Haike Road, Shanghai 201210, PR China

**Keywords:** muscle regeneration, fibro/adipogenic progenitors, muscle stem cells, proliferation, adipogenesis

## Abstract

Gli1 is a critical marker of diverse stem cell populations across multiple tissues and is essential for tissue regeneration. However, its functional relevance in skeletal muscle has remained largely unexplored. Here, we demonstrate that Gli1 primarily expressed in muscle stem cells (MuSCs) and fibro/adipogenic progenitors (FAPs) in skeletal muscle. Utilizing conditional knockout mouse models, we found that systemic loss of Gli1 impairs muscle regeneration; however, this effect is not attributable to MuSC-dependent mechanisms. Rather, conditional deletion of Gli1 in FAPs lead to significant regenerative impairment, characterized by aberrant FAP expansion and their enhanced adipogenic potential. *In vivo*, Gli1-deficient FAPs contributed to increased intramuscular adipocyte accumulation, while *in vitro* assays confirmed enhanced lipid droplet formation under adipogenic conditions. Mechanistically, Gli1 directly activates the transcription of the key metabolic enzyme indoleamine 2,3-dioxygenase 1 (Ido1), and inhibition or knockdown of Ido1 phenocopied the effects of Gli1 loss. Together, these findings uncover a previously unrecognized role for Gli1 in orchestrating muscle regeneration by modulating FAP fate and function, providing new insights into the cellular and molecular framework governing muscle repair.

## Introduction

Fibro/adipogenic progenitors (FAPs), as resident mesenchymal progenitors within muscle, are essential for maintaining muscle homeostasis and improve regeneration. Upon muscle injury, FAPs secrete growth factors that support the myogenic activity of muscle stem cells (MuSCs), thereby creating a microenvironment conducive to muscle tissue repair 1, 2. However, FAPs have a complex dual role in muscle repair, exerting both beneficial and detrimental effects depending on their quantity and functional dynamics. Excessive accumulation of FAPs has been linked to degenerative and pathological conditions such as aging, chronic diseases, and muscular dystrophies, leading to increased deposition of fibro-adipose tissues within muscle 2-8. Exploring the intrinsic mechanisms regulating FAP proliferation and function holds the potential to provide therapeutic insights for muscle-related diseases.

FAPs number are precisely regulated during muscle regeneration. Previous studies have shown that external stimuli, such as muscle injury, trigger a rapid influx of eosinophils that secrete interleukin-4, stimulating FAP proliferation and supporting myogenesis 9. FAPs are also responsive to platelet-derived growth factor, a secreted protein that promotes both FAP proliferation and collagen production 5. In general, FAPs exhibit a transient surge in proliferation following acute injury, with peak quantity occurring around the third to fourth day, followed by a return to baseline levels 8, 10. The depletion of FAPs results in significant impairments in muscle regeneration 11. Nevertheless, pathological conditions such as denervation and diabetes, which cause an abnormal increase in FAPs quantities, also hinder the repair process 5, 10. In a muscular dystrophy mouse model, excessive accumulation of FAPs was observed following injury, leading to intramuscular fat deposition, compromised muscle function, and impaired regeneration 12. FAPs exhibit remarkable heterogeneity 4, 13. Previous studies have identified FAP subpopulations, such as those marked by CD90 and Vcam1, which exhibit high proliferative capacities and contribute to muscle pathology 5, 14. These findings underscore the complexity of FAP biology, but the regulatory mechanisms governing their function remain poorly understood. Identifying additional FAP subpopulations with high proliferative potential and elucidating the mechanisms regulating their function could offer valuable new insights.

The Hedgehog (Hh) signaling pathway is an evolutionarily conserved signaling cascade that plays a pivotal role in muscle regeneration. Recent studies have demonstrated that the Hh pathway regulates the fate of FAPs 8, 15. Ectopic activation of the Hh pathway during chronic injury effectively inhibits the differentiation of FAPs into adipocytes *in vivo* 8. Gli1, as a downstream effector of Hh pathway, plays a role in a positive feedback loop by enhancing Gli activity 16. Increasingly studies highlighted the role of Gli1^+^ cells within muscle tissue, including MuSCs and FAPs 17, 18. In our previous study, we identified a population of Gli1^+^ MuSCs that functions as resident sentinels in muscle, rapidly responding to injury 17. However, the mechanisms by which Gli1 regulates muscle regeneration remain unclear.

In the present study, we demonstrated that FAPs were the primary Gli1-expressing cell population. We further confirmed the existence of Gli1^+^ FAPs using a dual recombinase-mediated genetic lineage tracing system. Conditional Gli1 knockout in FAPs resulted in excessive proliferation and impaired muscle regeneration. Moreover, Gli1 deletion in FAPs significantly enhanced their adipogenic capacity in both *in vivo* and *in vitro* models, suggesting that Gli1 negatively regulates FAPs adipogenesis. Mechanistically, we demonstrated that Gli1 directly binds to the promoter region of indoleamine 2,3-dioxygenase 1 (Ido1) and positively regulates its transcription, thereby modulate the proliferation and adipogenesis of FAPs. Pharmacological inhibition or siRNA-mediated knockdown of Ido1 significantly promoted FAP proliferation and adipogenesis. Our findings highlight the pivotal role of Gli1 in regulating muscle regeneration, emphasizing its critical function in controlling FAP proliferation and adipogenic differentiation. These insights provide potential therapeutic approaches for treating diseases related to skeletal muscle fibro-fatty degeneration.

## Materials and Methods

### Animals

Mice were housed and maintained in accordance with the guidelines of the Institutional Animal Care and Use Committee of the State Key Laboratory of Cell Biology, Shanghai Institute of Biochemistry and Cell Biology, Center for Excellence in Molecular Cell Science, University of Chinese Academy of Sciences, Chinese Academy of Sciences. Gli1^tm3(cre/ERT2)Alj^/J (Gli1^CreERT2^), B6.Cg-Ndor1Tg^(UBC-cre/ERT2)1Ejb^/1J (UBC^CreERT2^), B6.Cg-Pax7^tm1(cre/ERT2)Gaka^/J (Pax7^CreERT2^), B6.129S-Pdgfra^tm1.1(cre/ERT2)Blh^/J (PDGFRα^CreERT2^), B6.129P2-Gt(ROSA)26^Sortm1(DTA)Lky^/J (R26-DTA), and Gt(ROSA)26Sor^tm1(rtTA,EGFP)Nagy^ (R26-LSL-eGFP) mice were purchased from Jackson Laboratory. Wild type C57BL/6 was purchased from GemPharmatech. R26-RSR-tdT and PDGFRα^DreERT2^ mice were gifts from Dr. Bin Zhou 19. The C57BL/6JGpt-Gli1^em1Cflox^/Gpt (Gli1^f/f^) strain was constructed by flanking exons 3-11 of the Gli1 gene with loxP sites and generated by GemPharmatech. UBC^CreERT2^ and Gli1^f/f^ mice were crossed to generate UBC^CreERT2^; Gli1^f/f^ mice. Pax7^CreERT2^ and Gli1^f/f^ mice were crossed to generate Pax7^CreERT2^; Gli1^f/f^ mice. PDGFRα^CreERT2^ and Gli1^f/f^ mice were crossed to generate PDGFRα^CreERT2^; Gli1^f/f^ mice. Gli1^CreERT2^ and R26-LSL-eGFP mice were crossed to generate Gli1^CreERT2^; R26-LSL-eGFP mice. PDGFRα^DreERT2^ and R26-RSR-tdT were crossed to generate PDGFRα^DreERT2^; R26-RSR-tdT mice. Gli1^CreERT2^; R26-LSL-eGFP mice and PDGFRα^DreERT2^; R26-RSR-tdT mice were crossed to generate Gli1^CreERT2^; R26-LSL-eGFP; PDGFRα^DreERT2^; R26-RSR-tdT mice. Gli1^CreERT2^ and R26-DTA were crossed to generate Gli1^CreERT2^; R26-DTA mice. For the abbreviations used in the context of transgenic mouse models, R26 refers to the Rosa26 locus, a common genomic safe harbor for transgene insertion; LSL (LoxP-Stop-LoxP) and RSR (Rox-Stop-Rox) refer to transcriptional stop cassettes flanked by loxP or rox recombination sites, respectively, recognized by Cre and Dre recombinases. DTA refers to diphtheria toxin subunit A, a cytotoxic protein that, when expressed in a cell, leads to irreversible inhibition of protein synthesis and subsequent cell death. All mice were kept in group housing (3-6 mice/cage) in a specific pathogen-free (SPF) facility with controlled environmental conditions of temperature (20-25 °C), humidity (40-70%) and lighting (a 12 h light/dark cycle). Adult mice aged 8-10 weeks were used for all experiments. Control and experimental mice were littermate.

### Animal procedures

To induce specific gene expression or knockout, 8-week-old UBC^CreERT2^;Gli1^f/f^, Pax7^CreERT2^; Gli1^f/f^, PDGFRα^CreERT2^; Gli1^f/f^, Gli1^CreERT2^; R26-LSL-eGFP, PDGFRα^DreERT2^; R26-RSR-tdT, Gli1^CreERT2^; R26-LSL-eGFP; PDGFRα^DreERT2^; R26-RSR-tdT, Gli1^CreERT2^; R26-DTA mice were injected intraperitoneally with 20 mg/mL Tamoxifen (TAM, dissolved in corn oil) for 3 consecutive days with the total dose of 4 mg TAM per 20 g body weight. Mice with intraperitoneal injection of corn oil were considered as vehicle control.

For muscle cardiotoxin (CTX) injury model, the tibialis anterior (TA) muscles were injected with CTX (Sigma-Aldrich). Briefly, 50 µL of 10 µM CTX was injected at five injections site of TA muscles using 30G needles. The time points for sample collection were selected based on temporal dynamics of skeletal muscle regeneration following acute injury. In this study, muscle samples subjected to CTX injury were collected at 7 days post-injury (dpi), a time point when inflammation typically resolves and most injured muscle enters the regeneration phase.

For muscle glycerol injury model, the TA muscle was injected with 50 μL 50% vol/vol glycerol (Sigma; #G5516) in 1×PBS was injected at five injections sites of TA muscles using 30G needles. Muscle samples from the glycerol injury model were harvested at 9 dpi, when significant lipid accumulation is typically observed.

For Ido1 inhibitor injection, epacadostat was quantitatively dissolved in PEG300, followed by sonication to ensure complete dissolution. Normal saline was then incorporated to yield a final concentration of 15 mg/mL. Mice were administered the compound via injection at a dose of 100 mg/kg.

### FACS and isolation of FAPs

For isolation of FAPs by FACS, after mice were euthanized, hindlimb skeletal muscles were collected, finely chopped, and digested using 700 U/mL Collagenase type 2 (Worthington) in Wash medium (Ham's F-10 supplemented with 1% penicillin-streptomycin and 10% horse serum) for 60 min in a shaking water bath at 37 °C. The digested muscle was washed twice with Wash medium and centrifuged at 500×g for 5 min at 4 °C. A second digestion was performed with 1000 U/mL Collagenase type II and 1.1 U/mL Dispase II in Wash medium for 30 min in a shaking water bath at 37 °C. After digestion, the tissue was passed through a 20G needle three times to further dissociate the cells, followed by filtration through 70 μm and 40 μm cell strainers to obtain a s cell suspension suitable for FACS analysis.

Cell suspension was then stained with FITC anti-mouse CD31, FITC anti-mouse CD45, APC anti-mouse Sca1 antibodies. The cells were incubated with antibodies for 60 min on ice and subsequently washed with cold wash medium. DAPI was used for viable cell gating to exclude dead cells. CD31 was used to exclude endothelial cells and CD45 was used to exclude immune cells. FACS was performed using FACS Aria III (BD Biosciences) by gating for CD45^-^CD31^-^Sca1^+^ cells to isolate FAPs.

### FAPs culture

FACS-isolated FAPs were cultured in DMEM supplemented with 20% FBS, 1% Penicillin-Streptomycin, and 2.5 ng/mL bFGF. Adipogenic differentiation was initiated by culturing cells in adipogenic induction medium, composed of DMEM, 10% FBS, 0.5 mM IBMX, 0.25 μM dexamethasone, and 10 μg/mL insulin, for 3 days. Subsequently, cells were maintained in adipogenic maintenance medium containing DMEM, 10% FBS, and 10 µg/mL insulin. Osteogenic differentiation was induced using the osteogenic stimulatory kit (STEMCELL Technologies, #05504), and chondrogenic differentiation was induced using the chondrogenic differentiation kit (STEMCELL Technologies, #05455). All cells were fixed with 4% paraformaldehyde (PFA) at room temperature for 20 min, followed by Oil red O, alkaline phosphatase (ALP), and Alcian blue staining.

For Oil red O staining, cells were first covered with the staining wash solution for 20 seconds. The wash solution was then removed, and the Oil red O working solution (Beyotime, C0158S) was added for 20-30 min of staining. After staining, the solution was removed, and the cells were washed with PBS. An appropriate volume of PBS was added to cover the cells before imaging.

For ALP staining, cells were incubated with BCIP/NBT ALP staining solution (Beyotime, C3206) for 30 min. The staining solution was then removed, and the cells were washed with PBS. After washing, PBS was added to cover the cells prior to imaging.

For Alcian blue staining, the Alcian blue staining solution (Beyotime, C0153S) was applied to cover the cells for 40 min. After staining, the solution was removed, and the samples were stored in 70% ethanol for subsequent examination under a light microscope.

Proliferating FAPs were labeled with 5-Ethynyl-2'-Deoxyuridine (EdU) at a concentration of 5 ng/µL for 6 hours at the end of the culture period. EdU incorporation was detected using the Click-iT EdU Imaging Kit (Invitrogen, C10637) according to the manufacturer's instructions.

### Immunofluorescence staining and imaging

Freshly isolated TA muscles from Gli1^CreERT2^; R26-LSL-eGFP and Gli1^CreERT2^; R26-LSL-eGFP; PDGFRα^DreERT2^; R26-RSR-tdT were fixed in 4% PFA at 4 ºC for 60 min. After fixation, tissues were washed three times in PBS, dehydrated in 30% sucrose overnight at 4 ºC, embedded in OCT and flash frozen using liquid nitrogen. Samples from UBC^CreERT2^; Gli1^f/f^, Pax7^CreERT2^; Gli1^f/f^, PDGFRα^CreERT2^; Gli1^f/f^, and Gli1^CreERT2^; R26-DTA were not subjected to fixation and dehydration. The tissues were directly embedded in OCT and flash frozen using liquid nitrogen. 10 µm sections were collected on glass slides and stored at -20 °C. For immunofluorescence staining, the slides were put in a fume hood to air dry, followed by a brief PBS wash to remove OCT. For unfixed samples, a 20-min fixation in 4% PFA was required before proceeding with the same protocol as the fixed samples. All samples were permeabilized in 0.2% PBST for 20 min and blocked in 1% BSA for 1 h. Then, slides were incubated with primary antibodies overnight at 4 ºC in the dark. The primary antibodies used in this study are listed in Supplementary Table 1. On the following day, the slides were washed in PBS 3 times in PBS for 10 min each to remove unbound primary antibodies, followed by incubation with fluorescence-conjugated secondary antibodies and DAPI (4',6-diamidino-2-phenylindole, Sigma, D9542) for 60 min at room temperature in the dark. After washing 3 times in PBS for 5 min each to remove secondary antibodies, sections were mounted with Aqua-Poly/mount (Polysciences). For detecting eMyHC, a tyramide staining strategy was employed using the TSA Kit according to the manufacturer's instructions (Akoya). Images were acquired using Olympus fluorescence microscope VS120, FV4000, and Leica TCS SP8 confocal microscope. ImageJ software was used to analyze the collected images.

### CUBIC clearing

The CUBIC protocol was performed as previously reported 20, 21. Briefly, freshly isolated EDL muscles from Gli1^CreERT2^; R26-LSL-eGFP; PDGFRα^DreERT2^; R26-RSR-tdT mice at 5 days post-TAM induction were fixed in 4% PFA at 4 ºC for 24 h, followed by wash with PBS. The fixed EDL were immersed in 5 mL of 50% (v/v) CUBIC-L reagent (a 1: 1 mixture of water and CUBIC-L) for 1 day, and further immersed in 5 mL of CUBIC-L reagent for 5 days. After washing with PBS for 1 day, the EDL were immersed in 5 mL of CUBIC-R+ reagent for 4 days. Fluorescence images of EDL were acquired using Olympus FV4000 confocal microscope.

### Western blot

To detect Gli1 in UBC^CreERT2^; Gli1^f/f^ and Gli1^f/f^ mice, hind limb muscles from both mouse strains were lysed in RIPA buffer containing a 1× Protease Inhibitor Cocktail. The lysates were centrifuged at 10,000 g for 15 min at 4 ºC to remove insoluble material. Protein concentration was then determined using the BCA Protein Assay Kit. The supernatants were subsequently boiled in SDS loading buffer, and proteins were analyzed by Western blot using the indicated antibodies. Primary antibodies used in this study are listed in Supplementary Table 1.

### RNA-sequencing and real-time quantitative PCR

Total RNA was extracted from FAPs during both the proliferation and adipogenic differentiation stages using Trizol reagent (Invitrogen). Reverse transcription was conducted using the HiScript® III RT SuperMix for qPCR with gDNA wiper, following the manufacturer's protocol (Vazyme). RNA integrity was assessed using the Bioanalyzer 2100 system (Agilent). Messenger RNA was purified and fragmented, followed by cDNA synthesis. The library was prepared through end repair, adapter ligation, and amplification, then quantified and checked for size distribution. After library quality control, it is pooled and subjected to Illumina sequencing to produce 150 bp paired-end reads (Novogene, Tianjin, China). Then, RNA-seq reads were aligned to the reference genome using HISAT2 (version 2.0.5). Differential expression was assessed using DESeq2. GO annotation and KEGG pathway enrichment analysis were performed based on DAVID online database 22. For RT-qPCR, gene expression was quantified using AceQ Universal SYBR qPCR Master Mix (Vazyme), and the 2^-ΔΔCt^ method was applied for data analysis. RT-qPCR results were normalized to the housekeeping gene *Gapdh*. The primers used in this study are provided in Supplementary Table 2.

### Luciferase reporter assay

The plasmid pGL3-basic (Promega), a promoter-less luciferase plasmid, was served as a negative control (pGL3-vector), and the pGL3 plasmid containing 8×Gli1 binding site was served as a positive control (pGL3-8×BS). Cells were transfected with negative control plasmids, positive control plasmids, or a luciferase reporter construct containing Ido1 responsive regions (position -587 to +80 relative to the transcription starting site). Thirty-six hours after transfection, cells were harvested and luciferase activity was measured using a dual-luciferase reporter assay system (Promega, USA). Renilla luciferase activity was normalized based on firefly luciferase activity.

### Statistics and reproducibility

All data were collected from multiple independent biological samples and are presented as mean ±standard error of the mean (SEM). Statistical analyses were performed using unpaired one-sided Student's *t*-tests to compare differences between two groups, utilizing GraphPad Prism 8.0 software. Quantitative analyses of all immunofluorescence stainings were conducted using ImageJ software (version 1.53t). This included the assessment of myofiber cross-sectional area based on laminin staining, as well as the quantification of eMyHC⁺, perilipin⁺, EdU⁺, and PDGFRα⁺ cell populations. In addition, ImageJ was employed to quantify the staining areas of Oil Red O, ALP, and Alcian blue. All *in vitro* and *in vivo* experiments were independently performed with at least three biological replicates. A significance threshold was set at *P* < 0.05. The sample size (“n”), representing the number of biological replicates, is indicated within the manuscript. Mice were randomly assigned to experimental groups, and investigators analyzing the samples were blinded to group allocations.

## Results

### Gli1 regulates muscle regeneration through a MuSC-independent manner

**Figure 1:**
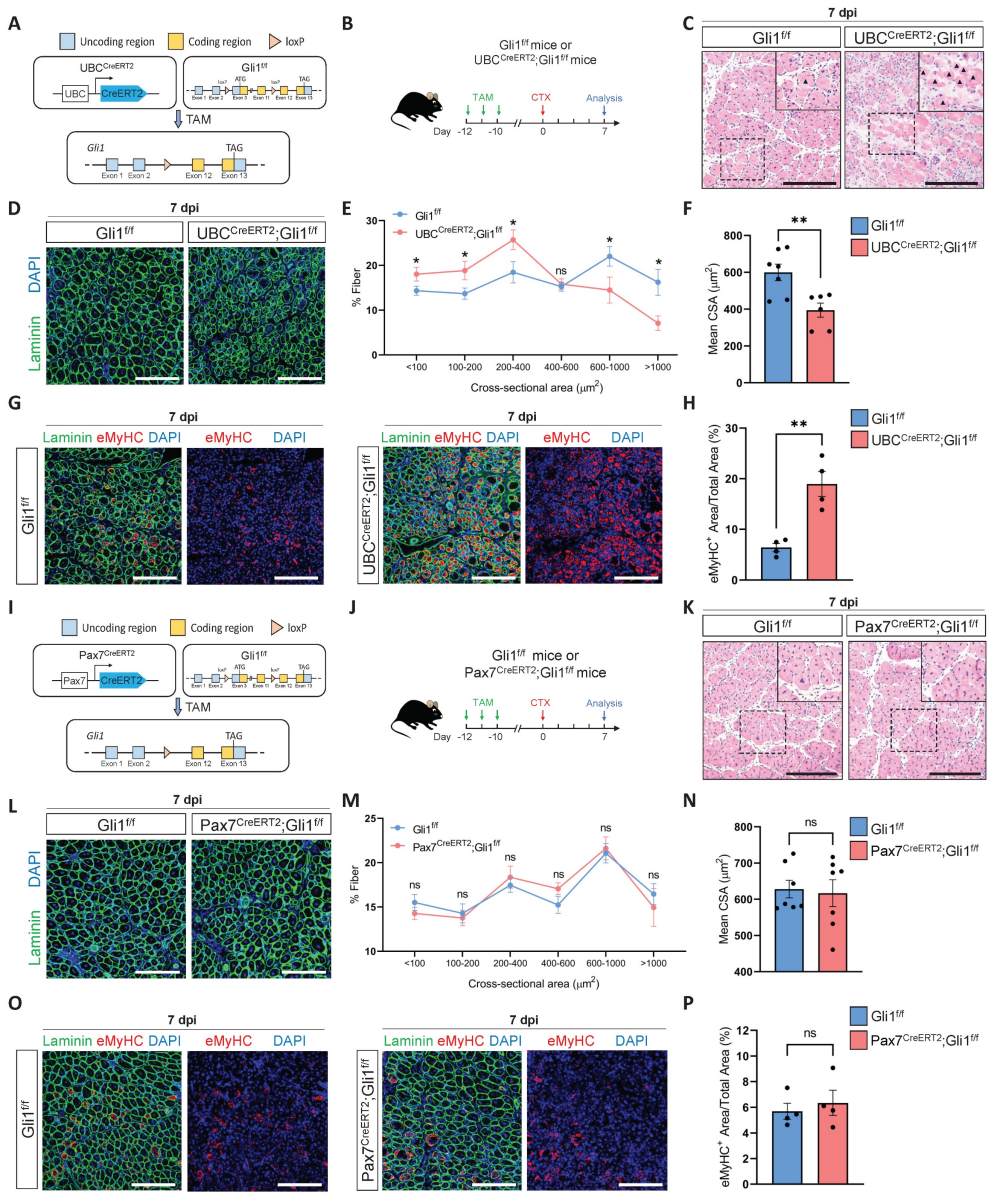
**Loss of Gli1 impairs muscle regeneration through a MuSC-independent manner. (A)** Schematic representation of the construction of UBC^CreERT2^;Gli1^f/f^ mice.** (B)** Scheme of the experimental strategy for UBC^CreERT2^;Gli1^f/f^ mice. **(C)** H&E staining of TA muscle sections from UBC^CreERT2^;Gli1^f/f^ mice at 7 dpi following CTX-induced injury. Scale bar, 200 μm. Black arrowheads indicate necrotic myofibers.** (D)** IF staining of laminin (green) and DAPI (blue) of TA muscle sections at 7 dpi following CTX-induced injury. Scale bar, 200 μm. **(E)** Quantification of the myofiber cross-sectional area (CSA, μm^2^) distribution (n = 6-7). Data are presented as mean ± SEM,^ *^P < 0.05, ns indicates not significant.** (F)** Quantification of the mean CSA (n = 6-7). Data are presented as mean ± SEM,^ **^P < 0.01. **(G)** IF staining of eMyHC (red), laminin (green), and DAPI (blue) in TA muscle sections at 7 dpi following CTX-induced injury. Scale bar, 200 μm. **(H)** Quantification of the percentage of eMyHC^+^ area within the total area (n = 4). Data are presented as mean ± SEM,^ **^P < 0.01. **(I)** Schematic representation of the construction of Pax7^CreERT2^;Gli1^f/f^ mice. **(J)** Scheme of the experimental strategy for Pax7^CreERT2^;Gli1^f/f^ mice. **(K)** H&E staining of TA muscle sections from Pax7^CreERT2^;Gli1^f/f^ mice at 7 dpi following CTX-induced injury. Scale bar, 200 μm. **(L)** IF staining of laminin (green) and DAPI (blue) in TA muscle sections at 7 dpi following CTX-induced injury. Scale bar, 200 μm. **(M)** Quantification of the myofiber CSA distribution (n = 7). Data are presented as mean ± SEM. ns indicates not significant. **(N)** Quantification of the mean CSA (n = 7). Data are presented as mean ± SEM, ns indicates not significant. **(O)** IF staining of eMyHC (red), laminin (green), and DAPI (blue) in TA muscle sections at 7 dpi following CTX-induced injury. Scale bar, 200 μm. **(P)** Quantification of the percentage of eMyHC^+^ area within the total area (n = 4). Data are presented as mean ± SEM, ns indicates not significant. All numbers (n) are biologically independent experiments.

To elucidate the role of Gli1 in muscle regeneration, we first generated a Gli1^f/f^ mice stain. The conditional Gli1 allele in this model allows for Cre-recombinase-mediated deletion spanning exons 3-11, thereby effectively disrupting Gli1 expression (Supplementary Figure 1A-B). Then, we crossed Gli1^f/f^ mice with UBC^CreERT2^ mice to obtain UBC^CreERT2^; Gli1^f/f^ mice, allowing for systemic ablation of Gli1 upon tamoxifen (TAM) administration (Figure 1A). To inactivate the Gli1 gene in adult UBC^CreERT2^; Gli1^f/f^ mice, we administered TAM at a concentration of 20 mg/mL to 2-month-old UBC^CreERT2^; Gli1^f/f^ mice and Gli1^f/f^ controls for 3 consecutive days, delivering a total dose of 4 mg TAM per 20 g body weight. Ten days post-TAM injection, RT-qPCR and Western blot analyses of hindlimb skeletal muscles from UBC^CreERT2^; Gli1^f/f^ mice revealed a significant decrease in both Gli1 mRNA and protein levels (Supplementary Figure 1C-D). To evaluate the role of Gli1 in muscle regeneration, skeletal muscle injury was induced in both UBC^CreERT2^; Gli1^f/f^ and control mice by intramuscular injection of cardiotoxin (CTX) into the tibialis anterior (TA) muscle, with muscle regeneration assessed 7 dpi (Figure 1B). Histological analysis revealed a significant impaired in muscle regeneration in UBC^CreERT2^; Gli1^f/f^ mice, characterized by persistent necrotic muscle fibers as shown by H&E staining (Figure 1C). Consistently, immunofluorescence (IF) staining for the immune cell markers CD45 and F4/80 also revealed markedly increased infiltration of inflammatory cells in Gli1-deficient muscles compared to control mice (Supplementary Figure 1E-H). Analysis of the mean cross-sectional area (CSA) and its frequency distribution demonstrated that UBC^CreERT2^; Gli1^f/f^ mice exhibited a reduced average CSA, with a marked shift in muscle fiber size distribution toward smaller fibers (Figure 1D-F). Embryonic myosin heavy chain (eMyHC), a robust marker of regeneration, is transiently expressed in regenerating muscle fibers during the early stages of injury 23, 24. In UBC^CreERT2^; Gli1^f/f^ mice, the regenerated TA muscle exhibited significantly higher proportion of eMyHC^+^ fibers at 7 dpi, compared to the low levels observed in control mice (Figure 1G-H). These findings suggest that Gli1 deletion disrupts the normal progression of muscle regeneration.

MuSCs are indispensable for muscle regeneration 25. Our previous findings identified Gli1 as a marker for a subpopulation of MuSCs with potent regenerative capabilities 17. To investigate whether Gli1 functions directly in MuSCs, we generated a mouse model with the conditional knockout of Gli1 specifically in MuSCs by crossing Pax7^CreERT2^ mice with Gli1^f/f^ mice (Figure 1I). The efficiency of Gli1 gene deletion in MuSCs was assessed by RT-qPCR, revealing a deletion efficiency of over 90% (Supplementary Figure 1I). Ten days after completing a 3-day TAM treatment, muscle regeneration was induced by CTX injection into the TA (Figure 1J). Surprisingly, ablation of Gli1 in MuSCs did not impair muscle regeneration. Histological analysis and IF staining revealed that Pax7^CreERT2^; Gli1^f/f^ mice exhibited similar morphology (Figure 1K), inflammatory cell infiltration (Supplementary Figure 1J-M), CSA distribution (Figure 1L-N), and the proportion of eMyHC^+^ fibers (Figure 1O-P) compared to Gli1^f/f^ controls. Taken together, these results indicate that Gli1 functions solely as a marker in MuSCs, with its deletion having no direct impact on muscle regeneration.

### Identification and multimodal validation of Gli1^+^FAPs

**Figure 2:**
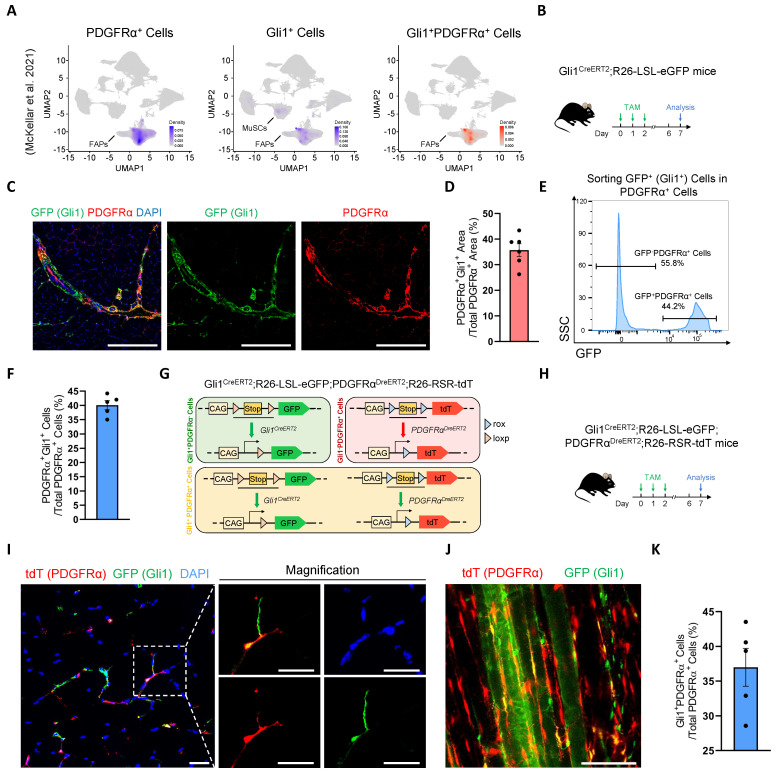
** Identification and characterization of Gli1^+^FAPs. (A)** UMAP of Gli1^+^PDGFRα^+^ cells in muscle analyzed from publicly available single-cell RNA sequencing (scRNA-seq) data. **(B)** Scheme of the experimental strategy for Gli1^CreERT2^;R26-LSL-eGFP mice. **(C)** IF staining of PDGFRα (red), GFP (green), and DAPI (blue) in TA muscle sections from Gli1^CreERT2^;R26-LSL-eGFP mice. Scale bar, 200 μm. **(D)** Quantification of the percentage of Gli1^+^PDGFRα^+^ area within the PDGFRα^+^ region (n = 6). Data are presented as mean ± SEM.** (E)** FACS analysis of GFP^+^ (Gli1^+^) cells within the FAP population in Gli1^CreERT2^;R26-LSL-eGFP mice. **(F)** Quantification of Gli1^+^PDGFRα^+^ cells within PDGFRα^+^ cells by FACS (n = 5). Data are presented as mean ± SEM. **(G)** Scheme of the dual-recombinase genetic lineage-tracing strategy. **(H)** Scheme of the experimental strategy for Gli1^CreERT2^;R26-LSL-eGFP;PDGFRα^DreERT2^;R26-RSR-tdT mice. **(I)** IF staining of tdT (red), GFP (green), and DAPI (blue) in TA muscle sections from Gli1^CreERT2^;R26-LSL-eGFP;PDGFRα^DreERT2^;R26-RSR-tdT mice. Scale bar, 25 μm.** (J)** IF staining of tdT (red) and GFP (green) in CUBIC-cleared EDL muscles from Gli1^CreERT2^;R26-LSL-eGFP;PDGFRα^DreERT2^;R26-RSR-tdT mice. Scale bar, 100 μm. **(K)** Quantification of Gli1^+^PDGFRα^+^ cells within PDGFRα^+^ cells in the CUBIC samples (n = 5). Data are presented as mean ± SEM. All numbers (n) are biologically independent experiments.

Our previous study demonstrated that Gli1 is expressed not only in MuSCs but also in other muscle cell types, with particularly high expression in FAPs 17. Combined with our findings that Gli1 regulates muscle regeneration in a MuSC-independent manner, we hypothesized that Gli1 may play a critical role in FAPs. Given the well-established importance of FAPs in muscle regeneration and their inherent heterogeneity, investigating whether Gli1 marks a FAP subpopulation and further exploring the role of Gli1 within this subpopulation could provide valuable insights into the mechanisms underlying muscle regeneration and tissue homeostasis. To test this hypothesis, we first analyzed a large-scale integration of publicly available single-cell RNA sequencing (scRNA-seq) data 26. As previous studies have demonstrated that PDGFRα expression is highly enriched and largely restricted to the FAP population in adult skeletal muscle 1, 4, we used PDGFRα as a marker to identify FAPs in single-cell RNA sequencing datasets and investigated its co-expression with Gli1. The results confirmed that, in addition to its expression in MuSCs, Gli1 was predominantly localized to PDGFRα^+^ FAPs. (Figure 2A).

To further validate the presence of Gli1^+^ FAPs identified in the scRNA-seq data, Gli1^CreERT2^; R26-LSL-eGFP mice were treated with TAM for three consecutive days, and tissues were harvested five days post-induction for further analysis (Figure 2B). Using these mice, we performed IF staining for PDGFRα on TA muscles to quantify the co-localization of PDGFRα and Gli1. The IF staining results indicated that ~35% of FAPs exhibited Gli1 expression (Figure 2C-D). Meanwhile, we isolated other hind limb muscles and used flow cytometry (FACS) to analyze the proportion of Gli1-expressing cells within the CD45^-^CD31^-^PDGFRα^+^ population. FACS analysis revealed that ~40% of FAPs had Gli1 expression (Figure 2E-F, Supplementary Figure 2A).

To achieve a more accurate characterization of Gli1^+^FAPs within muscle and to exclude potential interference from MuSCs, other cell types, and staining inaccuracies, we utilized a dual recombinase-mediated genetic lineage tracing system to precisely label and track Gli1^+^FAPs. For this purpose, we used the PDGFRα^DreERT2^ knock-in mouse strain, and crossed it with the R26-Rox-Stop-Rox-tdT (R26-RSR-tdT) reporter mouse strain. FACS analysis confirmed a recombination efficiency of ~42% in PDGFR^DreERT2^ mice (Supplementary Figure 2B). Then, we generated Gli1^CreERT2^; R26-loxP-Stop-loxP-eGFP; PDGFRα^DreERT2^; R26-Rox-Stop-Rox-tdT (Gli1^CreERT2^; R26-LSL-eGFP; PDGFRα^DreERT2^; R26-RSR-tdT) mouse strain. In this system, Cre recombination driven by Gli1 led to GFP expression, while Dre recombination under the PDGFRα promoter resulted in tdT expression (Figure 2G). Therefore, Gli1^+^PDGFRα^+^ FAPs were labeled with both GFP and tdT by administering TAM to this mice model. Five days post-TAM induction, TA muscles were collected for IF staining, extensor digitorum longus (EDL) muscles were harvested for CUBIC tissue clearing, and the remaining muscles were processed for FACS analysis (Figure 2H). Confocal images from IF staining of TA cryosections clearly confirmed the presence of GFP^+^tdT^+^ (Gli1^+^PDGFRα^+^ FAPs) cells (Figure 2I). Additionally, IF staining of CUBIC-cleared EDL showed the spatial distribution of Gli1^+^PDGFRα^+^ FAPs cells in three-dimensional tissue (Figure 2J, Supplementary Video 1), with ~37% of cells labeled as GFP^+^tdT^+^ (Figure 2K). Taken together, these findings consistently demonstrate that Gli1 marks a FAP subpopulation across various experimental models.

### Loss of Gli1 in FAPs leads to defective muscle regeneration and excessive accumulation of FAPs following injury

**Figure 3:**
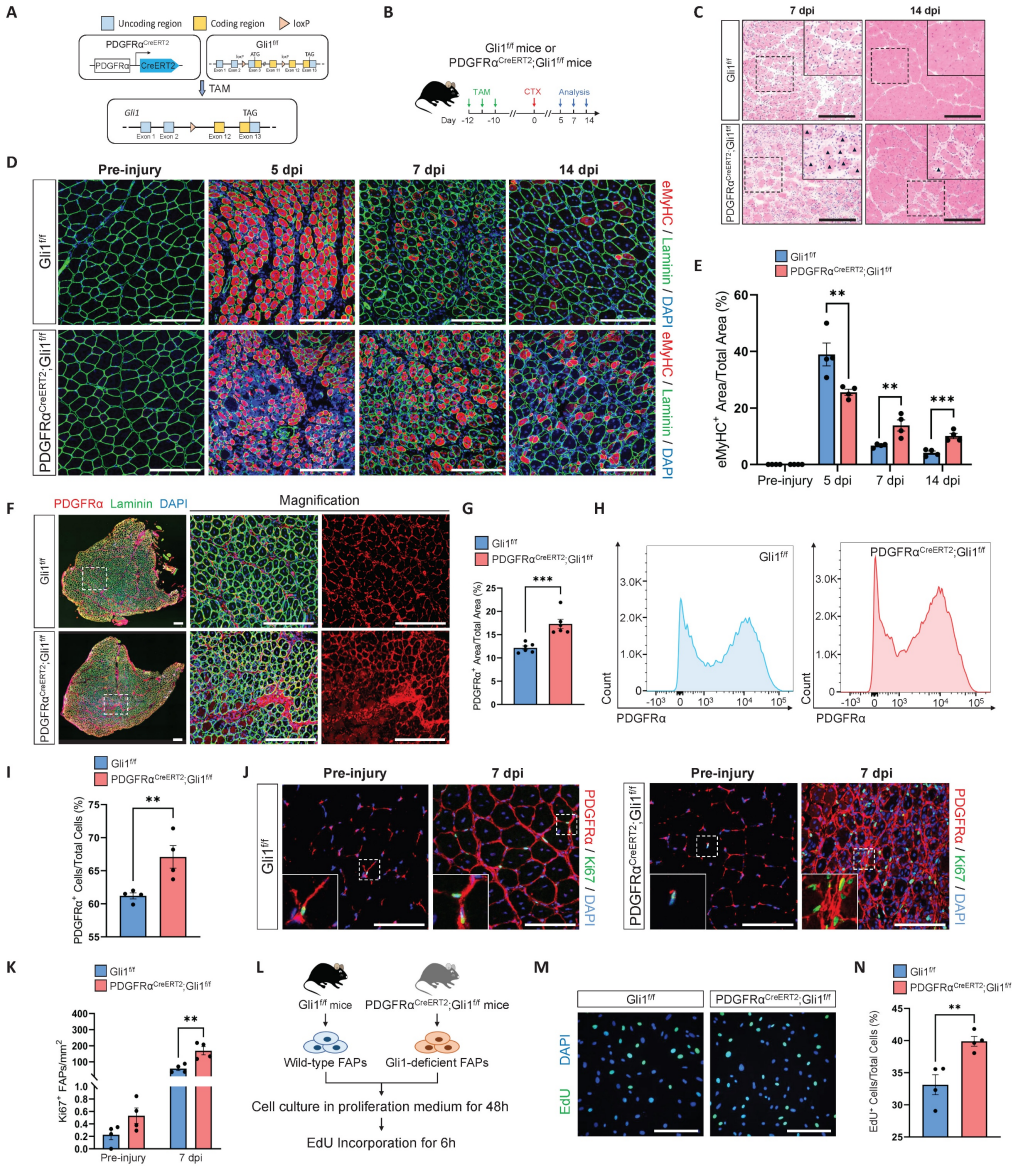
** FAP-specific loss of Gli1 significantly impairs muscle regeneration. (A)** Schematic representation of the construction of PDGFRα^CreERT2^;Gli1^f/f^ mice.** (B)** Scheme of the experimental strategy for PDGFRα^CreERT2^;Gli1^f/f^ mice. **(C)** H&E staining of TA muscle sections from PDGFRα^CreERT2^;Gli1^f/f^ mice and control mice at 7 dpi and 14 dpi following CTX-induced injury. Scale bar, 200 μm. Black arrowheads indicate necrotic myofibers. **(D)** IF staining of eMyHC (red), laminin (green), and DAPI (blue) of TA muscle sections from PDGFRα^CreERT2^;Gli1^f/f^ mice at pre-injury and at 5 dpi, 7 dpi, and 14 dpi following CTX-induced injury. Scale bar, 200 μm. **(E)** Quantification of eMyHC^+^ area within the total area (n = 4). Data are presented as mean ± SEM, ^**^P < 0.01,^ ***^P < 0.001.** (F)** IF staining of PDGFRα (red), laminin (green), and DAPI (blue) in TA muscle sections from PDGFRα^CreERT2^;Gli1^f/f^ mice at 7 dpi following CTX-induced injury. Scale bar, 250 μm.** (G)** Quantification of the percentage of PDGFRα^+^ area within the total area of TA muscle sections at 7 dpi following CTX-induced injury (n = 6). Data are presented as mean ± SEM,^ ***^P < 0.001. **(H)** FACS analysis of the proportion of FAPs in injured muscles from PDGFRα^CreERT2^;Gli1^f/f^ mice at 7 dpi following CTX-induced injury. **(I)** Quantification of the percentage of PDGFRα^+^ cells within total cells at 7 dpi following CTX-induced injury (n = 4). Data are presented as mean ± SEM,^ **^P < 0.01. **(J)** IF staining of PDGFRα (red), Ki67 (green), and DAPI (blue) in TA muscle sections from PDGFRα^CreERT2^;Gli1^f/f^ mice at pre-injury and at 7 dpi following CTX-induced injury. Scale bar, 100 μm. **(K)** Quantification of Ki67^+^ PDGFRα^+^ FAPs (n = 4). Data are presented as mean ± SEM, ^**^P < 0.01. **(L)** Scheme of the experimental strategy for EdU staining. **(M)** EdU staining of FAPs isolated and cultured for 48 h. Scale bar, 200 μm. **(N)** Quantification of the proportion of EdU^+^ cells (n = 4). Data are presented as mean ± SEM,^ **^P < 0.01. All numbers (n) are biologically independent experiments.

Given that Gli1 function is independent of MuSCs and is primarily localized in FAPs, we next investigated whether Gli1 is functional in FAPs. To this end, we generated PDGFRα^CreERT2^; Gli1^f/f^ mice to achieve FAP-specific deletion of Gli1 upon TAM induction (Figure 3A), followed by CTX injury (Figure 3B). PDGFRα^CreERT2^; Gli1^f/f^ mice exhibited a phenotype similar to UBC^CreERT2^; Gli1^f/f^ mice, marked by impaired clearance of necrotic muscle fibers (Figure 3C). Compared to control mice, PDGFRα^CreERT2^; Gli1^f/f^ mice exhibited a significant decrease in mean CSA, with a shift in the CSA distribution towards smaller muscle fibers (Supplementary Figure 3A-C). This was accompanied by a marked increase in inflammatory cell infiltration in the injured muscles from the PDGFRα^CreERT2^; Gli1^f/f^ mice compared to control mice (Supplementary Figure 3D-G). Notably, impaired muscle regeneration was observed at multiple time points post-injury, and regeneration remained significantly compromised at 14 dpi (Figure 3C-E, Supplementary Figure 3H-I).

FAPs undergo robust proliferation during the early stages of muscle injury, peaking around days 3-4 post-injury and gradually declining thereafter 10. To examine changes in FAPs numbers during injury, we performed IF staining for PDGFRα on TA muscles from PDGFRα^CreERT2^; Gli1^f/f^ mice and Gli1^f/f^ control mice. Of note, injured muscles from PDGFRα^CreERT2^; Gli1^f/f^ mice exhibited a significant accumulation of FAPs, substantially exceeding the levels observed in Gli1^f/f^ control mice (Figure 3F-G). This finding was further supported by FACS analysis, which confirmed a marked increase in the number of FAPs in limb muscles of PDGFRα^CreERT2^; Gli1^f/f^ mice compared to the Gli1^f/f^ control mice at 7 dpi (Figure 3H-I, Supplementary Figure 3J-K). We hypothesized that the accumulation of FAPs may be a result of excessive proliferation. To elucidate the role of Gli1 in regulating FAP proliferation, we performed IF staining using antibodies against Ki67 and PDGFRα to identify proliferating FAPs in injured muscle. The results revealed a significantly higher proportion of proliferating FAPs (Ki67^+^ FAPs) at 7 dpi in muscles of PDGFRα^CreERT2^; Gli1^f/f^ following injury (Figure 3J-K). These findings suggest that the loss of Gli1 may lead to excessive proliferation of FAPs.

Additionally, we isolated FAPs from PDGFRα^CreERT2^; Gli1^f/f^ and control mice for *in vitro* culture (Figure 3L). After 48 hours of culture in proliferation medium, 5-ethynyl-2′-deoxyuridine (EdU) was added for a 6-hour incubation, followed by IF staining. The results showed that loss of Gli1 in FAPs led to a pronounced increase in EdU incorporation (Figure 3M-N), indicating significantly elevated proliferative activity in Gli1-deficient FAPs.

### Gli1-mediated transcriptional regulation of Ido1 modulates FAP proliferation

**Figure 4:**
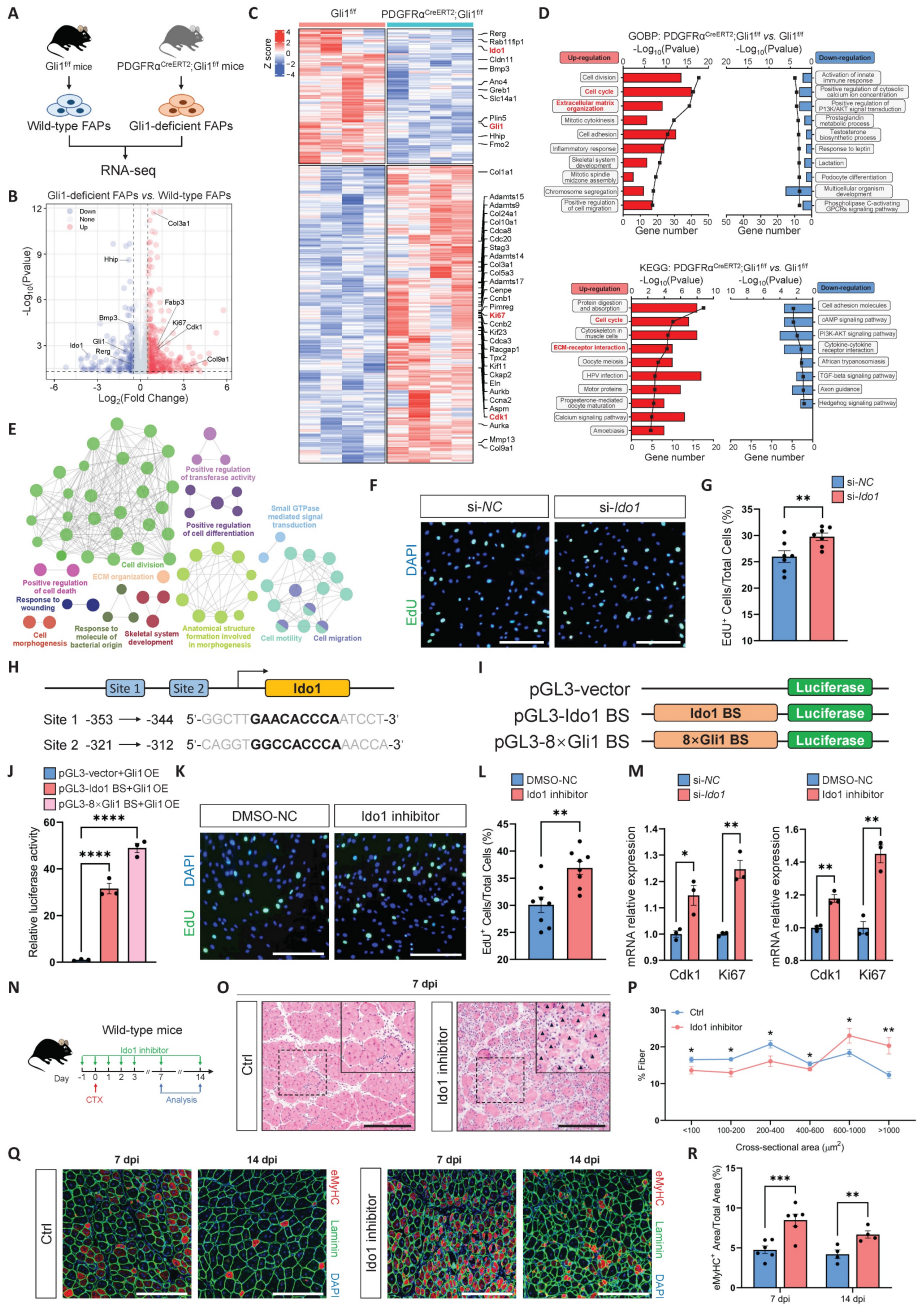
** Gli1 deficiency induces a proliferative transcriptional phenotype in FAPs. (A)** Scheme of the experimental strategy for RNA sequencing of FAPs. **(B)** Volcano plot of differentially expressed genes (DEGs) between Gli1-decifient FAPs and control FAPs. **(C)** Heatmap of DEGs showing transcriptional changes in FAPs. **(D)** GO and KEGG pathway enrichment analysis of DEGs.** (E)** Visualized functional enrichment network of GO terms by clueGO.** (F)** EdU staining of FAPs treated with si-*NC* or si-*Ido1*. Scale bar, 200 μm. **(G)** Quantification of the proportion of EdU^+^ cells after si-*NC* or si-*Ido1* treatment (n = 7). Data are presented as mean ± SEM,^ **^P < 0.01. **(H)** Scheme of the predicted Gli1 binding sites within the *Ido1* promoter region. **(I)** Schematic of the construction of luciferase reporter plasmids for testing *Ido1* promoter activity. BS, binding site. **(J)** Results of the luciferase reporter assays. Data are presented as mean ± SEM,^ ****^P < 0.0001. **(K)** EdU staining of FAPs treated with 0.5% DMSO or Ido1 inhibitor (50 nM) in DMSO. Scale bar, 200 μm. **(L)** Quantification of the proportion of EdU^+^ cells after DMSO or Ido1 inhibitor treatment (n = 8). Data are presented as mean ± SEM,^ **^P < 0.01. **(M)** Expression levels of *Cdk1* and *Ki67* in FAPs treated with si-*NC*, si-*Ido1*, 0.5% DMSO, or Ido1 inhibitor (50 nM). Data are presented as mean ± SEM,^ *^P < 0.05, ^**^P < 0.01. **(N)** Scheme of the experimental strategy for *in vivo* administration of the Ido1 inhibitor. **(O)** H&E staining of TA muscle sections from wild-type mice treated with or without Ido1 inhibitor at 7 dpi following CTX-induced injury. Scale bar, 200 μm. Black arrowheads indicate necrotic myofibers. **(P)** Quantification of the myofiber CSA distribution (n = 5). Data are presented as mean ± SEM,^ *^P < 0.05,^ **^P < 0.01. **(Q)** IF staining of eMyHC (red), laminin (green), and DAPI (blue) of TA muscle sections at 7 dpi and 14 dpi following CTX-induced injury. Scale bar, 200 μm. **(R)** Quantification of the eMyHC^+^ area within the total area (n = 4-6). Data are presented as mean ± SEM, ^**^P < 0.01,^ ***^P < 0.001. All numbers (n) are biologically independent experiments.

To compare the transcriptional differences between FAPs from PDGFRα^CreERT2^; Gli1^f/f^ mice and control mice, we performed bulk RNA sequencing (RNA-seq) on FAPs isolated from these mice (Figure 4A). RNA-seq data was examined using a DESeq2 package for differential expression analysis. In PDGFRα^CreERT2^; Gli1^f/f^ mice, Gli1-deficient FAPs showed significant changes in their transcriptional profile compared to Gli1^+^ FAPs from control mice. Our analysis identified 517 differentially expressed genes (DEGs), of which 356 were upregulated and 161 were downregulated (Figure 4B). Genes associated with proliferation (*Ki67*, *Ccna2*, *Ccnb1*, *Ccnb2*, *Cdk1*, *Cdca3*, *Caca8*, *Cdc20*, etc.), kinesin (*Cenpe*, *Kif11*, *Kif20b*, *Kif23*, etc.), collagen formation (*Col3a1*, *Col5a3*, *Col9a1*, *Col10a1*, *Col24a1*, etc.), and extracellular matrix (ECM) remodeling (*Adamts9*, *Adamts14*, *Adamts15*, *Adamts17*, *Mmp13*, etc.) were significantly upregulated in Gli1-deficient FAPs compared with FAPs from control mice (Figure 4C). Gene Ontology (GO) and KEGG pathway enrichment analysis showed that many of the significant terms were associated with the cell cycle and ECM formation (Figure 4D). A visualized functional enrichment network of GO terms further confirmed that cell division was the major category of DEGs (Figure 4E). Gene set enrichment analysis (GSEA) showed positive regulation of mTORC1 signaling and cyclin-dependent protein kinase activity in Gli1-deficient FAPs (Supplementary Figure 4A). Further validation by RT-qPCR confirmed the RNA-seq findings, showing downregulation of Hh signaling alongside upregulation of pathways related to ECM formation, cell proliferation, and mTORC1 signaling (Supplementary Figure 4B).

Given Gli1's role as a transcriptional activator, we hypothesized that its downstream target genes might mediate the regulation of FAP proliferation. To test this, we screened three downregulated genes (*Bmp3*, *Ido1*, and *Rerg*) with potential roles in regulating proliferation to evaluate their impact on FAP proliferation upon knockdown. Ido1 is a rate-limiting enzyme in the catabolism of tryptophan into kynurenine. Previous studies have shown that Ido1-mediated depletion of tryptophan in the local microenvironment suppresses immune cell proliferation 27, 28. To investigate the potential roles of Ido1 and two other candidate genes in regulating FAP proliferation, gene knockdown was achieved by transfecting FAPs with siRNAs targeting *Bmp3*, *Ido1*, and *Rerg*. Knockdown efficiency was validated using RT-qPCR (Supplementary Figure 4C). Subsequently, EdU incorporation assays were performed 24 hours post-transfection to assess proliferative activity. The results showed that only the knockdown of Ido1 significantly accelerated FAP proliferation (Figure 4F-G). In contrast, silencing of *Bmp3* and *Rerg* had no impact on FAP proliferation (Supplementary Figure 4D-E).

Further analysis revealed that Ido1 contains two Gli1 binding sites (-353 bp to -344 bp, and -321bp to -312 bp) upstream of its promoter region (Figure 4H). To further validate the regulatory role of Gli1 in Ido1 transcription, we constructed three reporter plasmids, including an empty vector as a negative control (pGL3-vector), a wild-type promoter plasmid containing the Gli1 binding site upstream of the Ido1 gene (pGL3-Ido1 BS), and a positive control plasmid with 8× Gli1 consensus binding sites (pGL3-8×BS) (Figure 4I). As expected, Gli1 overexpression significantly promoted luciferase activity in assay using pGL3-Ido1 BS and pGL3-8×BS plasmids (Figure 4J). Moreover, treatment with epacadostat, a potent and selective inhibitor of Ido1, significantly enhanced FAP proliferation at a concentration of 50 nM (Figure 4K-L), accompanied by the upregulation of *Cdk1* and *Ki67* (Figure 4M). These findings highlight the critical role of Ido1 as a Gli1-regulated gene that modulates FAP proliferation. To investigate the functional relationship between Gli1 and Ido1 at the phenotypic level, we hypothesized that inhibition of Ido1 would recapitulate the regenerative defects observed in Gli1-deficient mice. To test this, we administered an Ido1 inhibitor epacadostat to mice prior to CTX-induced muscle injury (Figure 4N). Consistent with our hypothesis, Ido1 inhibition significantly impaired muscle regeneration (Figure 4O-P). On day 14 post-injury, mice with Ido1 inhibitor administration exhibited a greater proportion of immature regenerating myofibers compared to controls, indicating impaired muscle repair (Figure 4Q-R).

To further elucidate the regulatory connection involving Gli1 and Ido1 in FAP proliferation, we examined the effects of Ido1 modulation in Gli1-deficient FAPs (Supplementary Figure 4F).

The results showed that Ido1 inhibition did not further enhance the elevated proliferative phenotype observed upon Gli1 deletion, thereby implying a shared underlying regulatory pathway (Supplementary Figure 4G-H). Conversely, Ido1 overexpression in the Gli1-deficient context significantly suppressed FAP proliferation (Supplementary Figure 4I-J). Notably, restoring Gli1 expression partially reduced proliferative activity, an effect abrogated by Ido1 inhibition (Supplementary Figure 4K-L). These findings collectively indicate that Ido1 acts downstream of Gli1 to restrain FAP proliferation.

### Loss of Gli1 in FAPs leads to lipid accumulation following muscle injury

**Figure 5:**
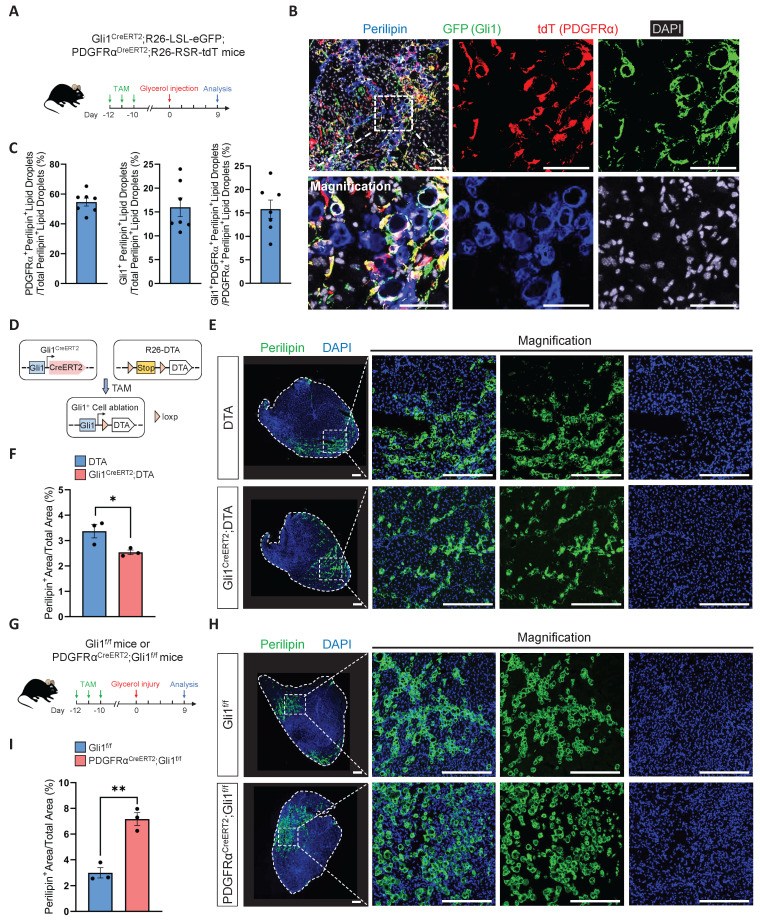
** FAP-specific loss of Gli1 significantly enhances the adipogenic potential of FAPs. (A)** Scheme of the experimental strategy for Gli1^CreERT2^;R26-LSL-eGFP;PDGFRα^DreERT2^;R26-RSR-tdT mice. **(B)** IF staining for tdT (red), GFP (green), perilipin (blue), and DAPI (gray) in TA muscle sections from Gli1^CreERT2^;R26-LSL-eGFP;PDGFRα^DreERT2^;R26-RSR-tdT mice following glycerol-induced injury. Scale bar, 50 μm. **(C)** Proportion of PDGFRα^+^perilipin^+^ lipid droplets out of total perilipin^+^ lipid droplets, Gli1^+^perilipin^+^ lipid droplets out of total perilipin^+^ lipid droplets, and Gli1^+^PDGFRα^+^perilipin^+^ lipid droplets out of PDGFRα^+^perilipin^+^ lipid droplets (n = 7). Data are presented as mean ± SEM.** (D)** Scheme of genetic ablation of Gli1^+^ cells using Gli1^CreERT2^;DTA mice. **(E)** IF staining for perilipin (green) and DAPI (blue) in TA muscle sections from Gli1^CreERT2^;DTA mice and control mice following glycerol-induced injury. Scale bar, 250 μm. **(F)** Quantification of the proportion of perilipin^+^ area (n = 3). Data are presented as mean ± SEM,^ *^P < 0.05. **(G)** Scheme of the experimental strategy for PDGFRα^CreERT2^;Gli1^f/f^ mice.** (H)** IF staining for perilipin (green) and DAPI (blue) in TA muscle sections from PDGFRα^CreERT2^;Gli1^f/f^ mice and control mice following glycerol-induced injury. Scale bar, 250 μm. **(I)** Quantification of the proportion of perilipin^+^ area (n = 3). Data are presented as mean ± SEM,^ **^P < 0.01. All numbers (n) are biologically independent experiments.

In our RNA-seq analysis, we found a significant upregulation of key genes (Acly, Fads3, Fasn, Srebp2) in the fatty acid synthesis pathway in Gli1-deficient FAPs compared to control FAPs (Supplementary Figure 5A-B). As resident mesenchymal stem cells in muscle, FAPs possess the capacity to differentiate into adipocytes, osteoblasts, and chondrocytes. Previous studies have shown that ciliary Hh signaling restricts injury-induced adipogenesis in FAPs 8. Based on these findings, we hypothesized that Gli1 may also play a role in adipogenesis. To test this hypothesis, we first performed glycerol-induced injury on Gli1^CreERT2^; R26-LSL-eGFP mice. In our study, we observed that ~25% of perilipin^+^ lipid droplets originated from Gli1^+^ cells at 9 dpi (Supplementary Figure 5C-D). To definitively exclude the contribution of non-FAPs to lipid droplet formation and investigate the specific contribution of Gli1^+^ FAPs to adipogenesis, we subsequently employed Gli1^CreERT2^; R26-LSL-eGFP; PDGFRα^DreERT2^; R26-RSR-tdT mice to trace the trajectory of Gli1^+^PDGFRα^+^ FAPs following glycerol-induced muscle injury (Figure 5A). At 9 dpi, IF staining showed that Gli1^+^PDGFRα^+^ FAPs contributed to ~15% of post-injury lipid droplet formation. (Figure 5B-C). Given that Gli1^+^ FAPs constitute approximately 35%-40% of the total FAP population but contribute only about 15%-25% to lipid droplet formation, these findings suggest that their adipogenic potential is relatively limited.

To further validate the contribution of Gli1^+^ FAPs to adipogenesis, we generated a Gli1^CreERT2^; R26-DTA mice. In this model, 50-60% Gli1^+^ cells were ablated upon TAM-induced Cre-mediated activation of diphtheria toxin A (DTA) expression (Figure 5D, Supplementary Figure 5E-F). Muscle regeneration in these mice was assessed following glycerol-induced injury, with TA muscles analyzed at 9 dpi. We observed a mild reduction in lipid accumulation in the Gli1^CreERT2^; DTA group compared to the DTA control group, suggesting that Gli1^+^ cells, including Gli1^+^ FAPs, play a limited role in adipogenesis (Figure 5E-F). Given the weak adipogenic capacity of Gli1^+^ FAPs, we hypothesize that Gli1 might also function to restrict the adipogenic potential of FAPs. To test this, we induced glycerol injury in PDGFRα^CreERT2^; Gli1^f/f^ mice (Figure 5G). The results showed a significant increase in adipose deposition following muscle injury when Gli1 was deleted in FAPs (Figure 5H-I), indicating that Gli1 acts as a suppressor of adipogenesis.

**Figure 6:**
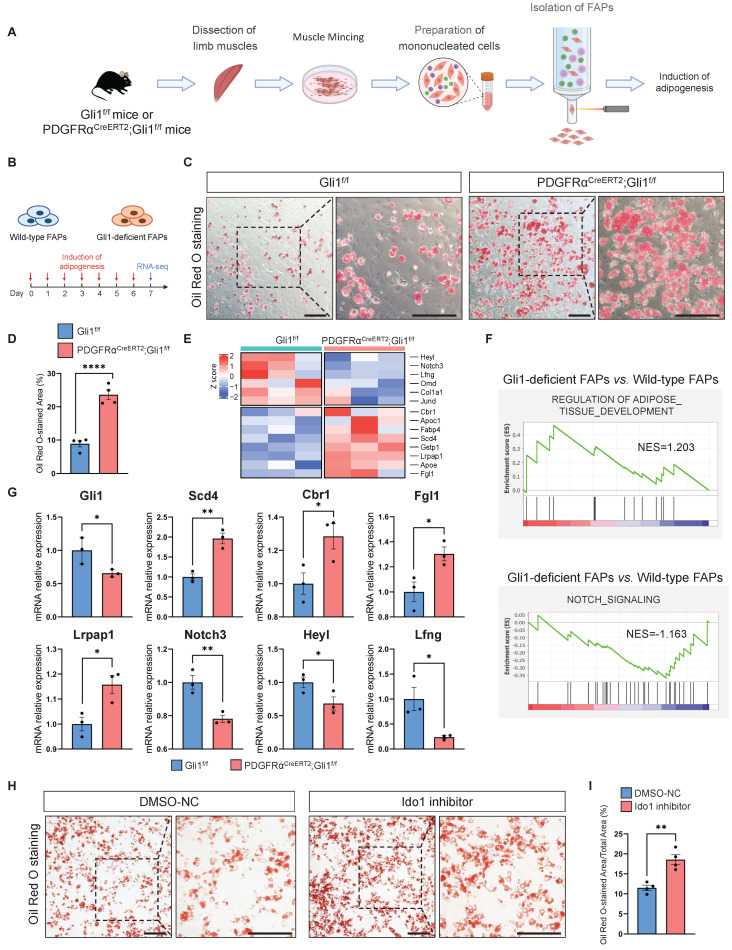
** Gli1 deficiency promotes adipogenesis of FAPs *in vitro*. (A)** Scheme of the experimental strategy for sorting FAPs using FACS. **(B)** Scheme of the culture strategy and RNA-seq of isolated FAPs.** (C)** Oil red O staining of FAPs from PDGFRα^CreERT2^;Gli1^f/f^ and control mice. Scale bar, 200 μm.** (D)** Quantification of the proportion of Oil red O-stained area (n = 4) between FAPs from PDGFRα^CreERT2^;Gli1^f/f^ and control mice. Data are presented as mean ± SEM,^ ****^P < 0.0001. **(E)** Heatmap of the representative genes related to adipogenesis and Notch signaling. **(F)** GSEA of positive regulation of adipose tissue development and negative regulation of Notch signaling. **(G)** Validation of DEGs by RT-qPCR (n = 3). Data are presented as mean ± SEM, ^*^P < 0.05, ^**^P < 0.01. **(H)** Oil red O staining of FAPs from wild-type mice treated with or without Ido1 inhibitor. Scale bar, 200 μm. **(I)** Quantification of the proportion of Oil red O-stained area (n = 4) between FAPs treat with or without Ido1 inhibitor. Data are presented as mean ± SEM,^ **^P < 0.01. All numbers (n) are biologically independent experiments.

To directly assess the impact of Gli1 on the differentiation potential of FAPs, we isolated FAPs from both PDGFRα^CreERT2^; Gli1^f/f^ mice and Gli1^f/f^ control mice using FACS (Figure 6A, Supplementary Figure 6A). We induced adipogenic, osteogenic, and chondrogenic differentiation by adding respective differentiation medium (Figure 6B, Supplementary Figure 6B). Our data demonstrated that ablation of Gli1 significantly increased adipogenesis (Figure 6C-D) while decreased osteogenesis in FAPs (Supplementary Figure 6C-D). Chondrogenic differentiation was not significantly affected (Supplementary Figure 6E-F). These findings were further validated by transcriptomic analysis, which revealed significant upregulation of adipogenesis-related genes (*Apoc1*, *Apoe*, *Cbr1*, *Fabp4*, *Fgl1*, *Gstp1*, *Lrpap1*, *Scd4*), and downregulation of osteogenic differentiation genes (*Col1a1*, *Jund*, *Omd*) and Notch signaling-related genes (*Heyl*, *Lfng*, *Notch3*) in Gli1-deficient FAPs (Figure 6E). Notably, Notch signaling is known to reduce the differentiation of FAPs into adipocytes *in vitro* and *in vivo* 29. GSEA analysis revealed an enrichment of adipogenic pathways and suppression of Notch signaling in Gli1-deficient FAPs (Figure 6F). These findings were supported by RT-qPCR, which confirmed the upregulation of adipogenic genes and downregulation of Gli1 and Notch-related genes (Figure 6G).

To investigate whether Ido1 affects the adipogenic potential of FAPs, we added 50 nM of the Ido1 inhibitor epacadostat to the culture medium of FAPs isolated from WT mice. After 72 hours of incubation, adipogenic differentiation was induced. The results showed that epacadostat significantly enhanced the adipogenic capacity of FAPs (Figure 6H-I). Taken together, these results suggest that Gli1 plays a crucial role in restricting the adipogenic capacity of FAPs. Furthermore, to validate the mechanistic connection between Gli1, Ido1, and adipogenesis, we treated Gli1-deficient FAPs with either an Ido1 inhibitor or Ido1 overexpression (Supplementary Figure 6G). Results indicated that inhibition of Ido1 in the Gli1-deficient background did not produce an additive effect (Supplementary Figure 6H-I), whereas Ido1 overexpression markedly attenuated the adipogenesis (Supplementary Figure 6J-K). Consistently, overexpression of Gli1 in Gli1-deficient FAPs partially suppressed their adipogenic potential, whereas subsequent treatment with an Ido1 inhibitor reversed this effect, supporting the role of Ido1 as a downstream mediator of Gli1 in regulating adipogenesis (Supplementary Figure 6L-M).

## Discussion

**Figure 7:**
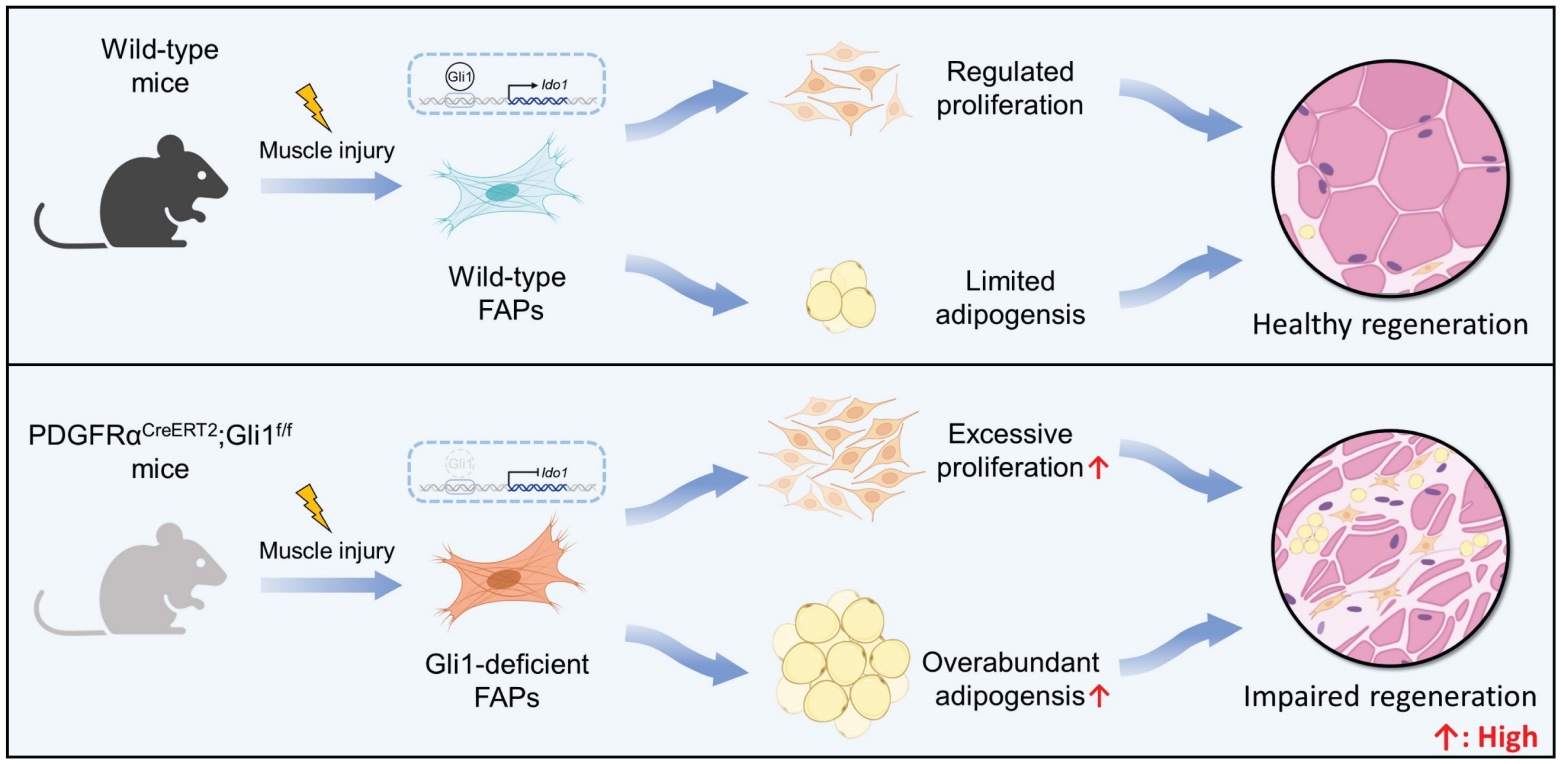
** Schematic illustrating the critical role of Gli1 in regulating FAP function.** Gli1 serves as a critical regulator of FAP activity in wild-type mice, maintaining a balanced state that prevents excessive proliferation and adipogenic differentiation. This regulation is essential for preserving muscle tissue integrity and supporting normal muscle regeneration. In contrast, downregulation or loss of Gli1 expression in FAPs leads to dysregulated proliferation and adipogenic differentiation through the modulation of Ido1, leading to intramuscular fat accumulation, disrupted muscle microenvironment, and impaired muscle regeneration.

FAPs are mesenchymal progenitors residing in muscle tissue that plays an essential role in maintaining tissue homeostasis and facilitating repair processes. Despite of that, under disease states and age-related conditions, overaccumulation and functional abnormalities of FAPs result in significantly intramuscular fat infiltration, ECM deposition, and impaired muscle regeneration 2, 4-8. However, the intrinsic mechanisms that govern FAP quantity and function dynamics remain unclear. Here, we demonstrate that Gli1 plays a crucial role in muscle repair following injury through a MuSC-independent but FAP-dependent mechanism. Employing genetic lineage tracing, we identify a distinct subpopulation of FAPs marked by Gli1 expression. Loss of Gli1 resulted in accelerated proliferation and adipogenesis of FAPs, severely disrupting muscle regeneration and promoting adipose tissue accumulation in injured muscles (Figure 7). These findings highlight the critical regulatory function of Gli1 in controlling FAP behavior and maintaining proper muscle regeneration.

The Hh pathway is a key regulator of muscle development during embryogenesis, and plays an essential role in the maintenance and regeneration of adult muscle. As a key downstream transcriptional activator of the Hh pathway, Gli1 has been identified as a marker of progenitors in diverse tissues and organs, including perivascular regions 30, growth plates 31, liver 32, and lung 27. In our previous study, we identified a population of primed MuSCs marked by Gli1 under homeostatic conditions. These cells exhibited rapid responsiveness to external stimuli and had robust regenerative potential, playing a critical role in skeletal muscle repair 17. These observations underscore the widespread distribution of Gli1-positive cells and their pivotal roles in a variety of biological processes. However, the function of Gli1 has been rarely studied. In this study, we found that the deletion of Gli1 in MuSCs did not impair muscle regeneration. Similar observations have been reported where genes serving as cellular markers were found to be dispensable for their cellular functions upon deletion. For instance, while Tcf21 marks cardiac fibroblasts and Gata6 marks pericardial macrophages, their deletion in the cardiac system did not alter cardiac fibrosis after myocardial infarction 33, 34. In this study, we demonstrated that Gli1 is expressed and functionally active in FAPs. These findings suggest that Gli1 may primarily serve as a marker of MuSC regenerative potential rather than as a direct regulator of their function.

FAPs contribute to muscle repair by secreting cytokines and ECM proteins that create a regenerative niche, allowing MuSCs to rebuild damaged muscle fibers. However, the intrinsic regulatory mechanisms by which FAPs respond to muscle injury remain poorly understood, and the role of Gli1 in this process is unclear. Previous studies on Gli1 in other cell types have demonstrated its functional diversity, with evidence showing that Gli1 can either promote or inhibit proliferation depending on the cellular context. For example, the loss of Gli1 leads to a transient increase in the proliferation of neural stem cells in the subventricular zone during the early stage of demyelination-induced disease 35. In the hematopoietic system, Gli1-deficient hematopoietic stem cells and progenitors show reduced levels of Cyclin D1, accompanied by a loss of proliferative capacity 36.

Our findings demonstrate that Gli1 marks a FAPs subpopulation and genetic ablation of Gli1 in FAPs leads to a significant increase in FAP proliferation and accumulation within the injured muscle. At the transcriptional level, we found that Gli1 regulates FAP function by modulating Ido1 expression. Ido1 is a rate-limiting enzyme that catalyzes the conversion of the essential amino acid tryptophan to kynurenines and has been extensively studied in tumor-related research. Ido1 and its metabolite kynurenine, are known to significantly suppress T cell proliferation. However, the function of Ido1 in non-tumor diseases remains largely unexplored. Consistent with our findings, studies have shown that IFN-γ inhibits mesenchymal cell proliferation through Ido1 activation 37. Besides, our observation that Gli1-deficient FAPs exhibited impaired osteogenic capacity is supported by evidence that inhibition of Ido1 in human mesenchymal stem cells significantly suppressed osteogenesis 38. These findings underscore the critical role of Gli1-Ido1 signaling in regulating both the proliferative and differentiation potential of FAPs.

During obesity and aging, skeletal muscle often exhibits increased deposition of fibro-adipose tissue, primarily originating from FAPs 39, 40. Understanding the regulatory mechanisms of FAP function offers promising targets for treating muscle dysfunction. Recent single-cell sequencing studies on young and aged hind limb muscles revealed that Gli1 expression is downregulated across various FAPs subtypes in aged muscle 41. Moreover, Gli1 expression was significantly downregulated in muscles of aged mice following injury 42. Aging is often associated with impaired muscle regeneration, and the downregulation of Gli1 in this context may underscore the critical importance of its regulatory functions. A time-course analysis revealed that Gli1 expression in the TA muscles peaked around days 3-4 post-injury, coinciding with the peak of FAP quantity 10, 42. We hypothesize that Gli1 is a critical factor in maintaining FAP stemness by limiting excessive adipogenic differentiation of FAPs, thereby preventing pathological fibro-adipose tissue deposition during injury. This hypothesis was confirmed by our dual recombinase-mediated intersectional genetic lineage tracing analysis. Compared to PDGFRα antibody staining, the lineage tracing system enables continuous tracking of the fate of originally labeled cells, regardless of changes in marker expression following differentiation. Through this approach, we found that Gli1⁺ FAPs exhibited a limited capacity for adipogenesis. Besides, loss of Gli1 significantly enhanced the adipogenic differentiation potential of FAPs, leading to increased adipose tissue deposition following muscle glycerol injury. These results highlight the critical role of Gli1 in restricting FAPs adipogenesis and provide novel insights into the mechanisms underlying muscular fat deposition.

In conclusion, our research identifies a previously unrecognized role for Gli1 and Ido1 in modulating FAPs function. We demonstrate that Gli1and Ido1 is essential for regulating FAP proliferation and adipogenesis. These findings suggest that therapeutic interventions targeting Gli1 and its downstream signaling pathways hold significant promising for attenuating stromal degeneration and adipose tissue accumulation associated with diseases.

## Supplementary Material

Supplementary figures and tables.

Supplementary video.
