# Supplementary material for: Gli1 regulates fibro/adipogenic progenitor function through modulation of Ido1 in muscle regeneration
Source: Int J Biol Sci. 2025 Sep 29;21(14):6197–214. doi: 10.7150/ijbs.116134 (PMC12594572; doi:10.7150/ijbs.116134)
Supplement: Supplementary file 1 — Supplementary figures and tables. [file ijbsv21p6197s1.pdf]

Supplementary Materials for

Gli1 regulates fibro/adipogenic progenitor function through modulation of Ido1 in muscle regeneration

Lili Han, Fengmin Zhang, Jujin Zhang, Xiaonan Li, Kunpeng Wang, Biao Liu, Jiawen Song, Xinyan Liu, Yun Qian, Kai Li, Yingnan Lei, Claudia Spits, Yi Chang, Chengle Zhuang, Zhen Yu, Yun Zhao*, Jiayin Peng*

Correspondence to: Jiayin Peng (jiayin.peng@sibcb.ac.cn), Yun Zhao (yunzhao@sibcb.ac.cn),

This PDF file includes:

Supplementary Figure 1-6

Supplementary Table 1-2

Supplementary Figure 1

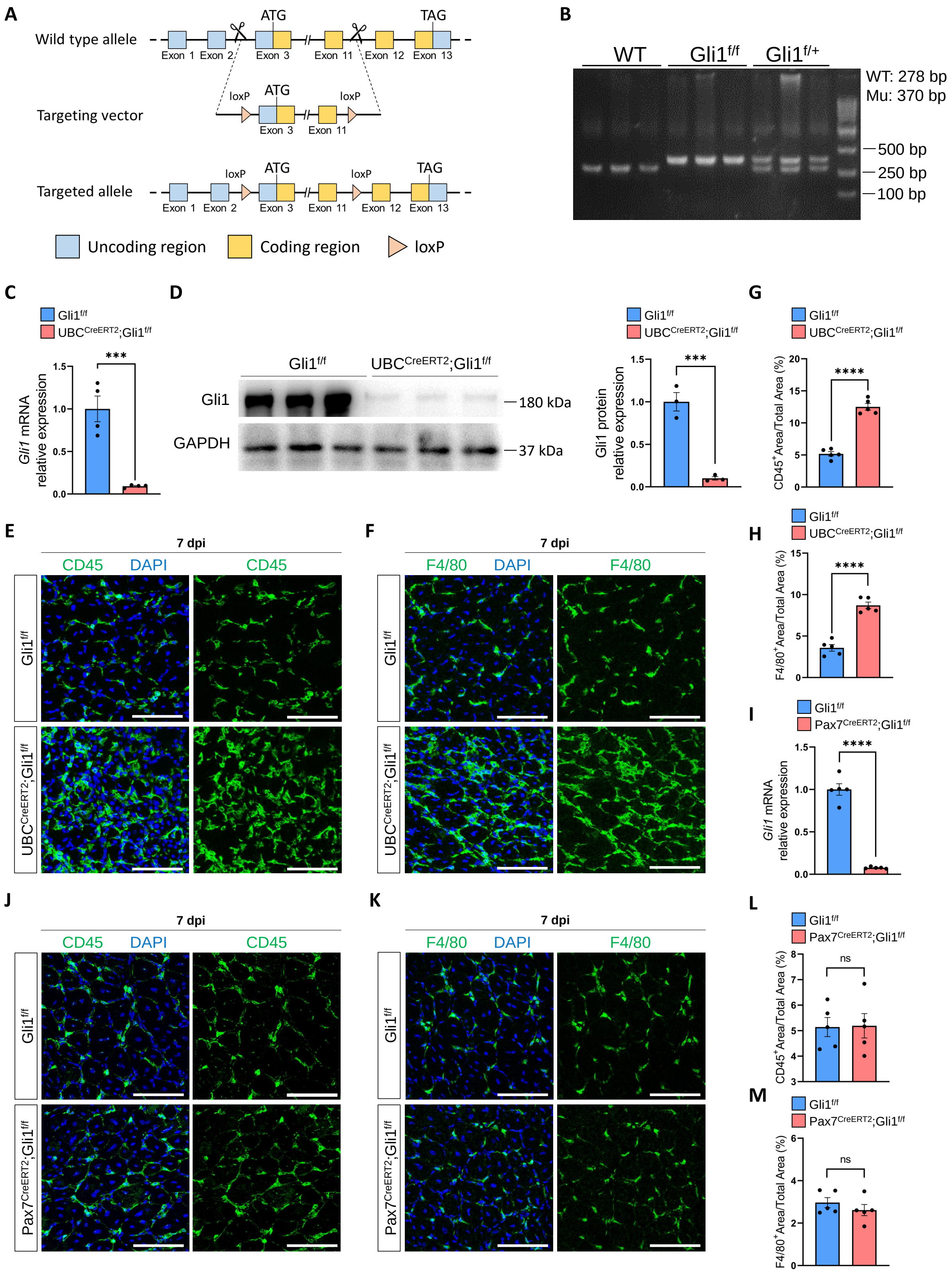

Supplementary Figure 1. Construction strategy of Gli1$^{f/f}$ mice. (A) CRISPR/Cas9 nuclease-mediated strategy for the construction of Gli1$^{f/f}$ mice. **(B)** Genotyping results of wild-type (WT), Gli1$^{f/f}$, and Gli1$^{f/+}$ mice. **(C)** RT-qPCR analysis of Gli1 expression levels in muscles from UBC^{CreERT2};Gli1$^{f/f}$ mice (n = 4). Data are presented as mean ± SEM, $^{***}P < 0.001$. **(D)** Western blot analysis of Gli1 expression levels (n = 3). Data are presented as mean ± SEM, $^{***}P < 0.001$. **(E)** IF staining of CD45 (green) and DAPI (blue) of TA muscle sections from UBC^{CreERT2};Gli1$^{f/f}$ mice at 7 dpi following CTX-induced injury. Scale bar, 100 µm. **(F)** IF staining of F4/80 (green) and DAPI (blue) of TA muscle sections from UBC^{CreERT2};Gli1$^{f/f}$ mice at 7 dpi following CTX-induced injury. Scale bar, 100 µm. **(G)** Quantification of the CD45^{+} area within the total area in CTX-injured TA muscles from UBC^{CreERT2};Gli1$^{f/f}$ mice (n = 5). Data are presented as mean ± SEM, $^{****}P < 0.0001$. **(H)** Quantification of the F4/80^{+} area within the total area in CTX-injured TA muscles from UBC^{CreERT2};Gli1$^{f/f}$ mice (n = 5). Data are presented as mean ± SEM, $^{****}P < 0.0001$. **(I)** RT-qPCR analysis of Gli1 expression levels in MuSCs from Pax7^{CreERT2};Gli1$^{f/f}$ mice (n = 5). Data are presented as mean ± SEM, $^{****}P < 0.0001$. **(J)** IF staining of CD45 (green) and DAPI (blue) of TA muscle sections from Pax7^{CreERT2};Gli1$^{f/f}$ mice at 7 dpi following CTX-induced injury. Scale bar, 100 µm. **(K)** IF staining of F4/80 (green) and DAPI (blue) of TA muscle sections from Pax7^{CreERT2};Gli1$^{f/f}$ mice at 7 dpi following CTX-induced injury. Scale bar, 100 µm. **(L)** Quantification of the CD45^{+} area within the total area in CTX-injured TA muscles from Pax7^{CreERT2};Gli1$^{f/f}$ mice (n = 5). Data are presented as mean ± SEM, ns indicates not significant. **(M)** Quantification of the F4/80^{+} area within the total area in CTX-injured TA muscles from Pax7^{CreERT2};Gli1$^{f/f}$ mice (n = 5). Data are presented as mean ± SEM, ns indicates not significant. All numbers (n) are biologically independent experiments.

Supplementary Figure 2

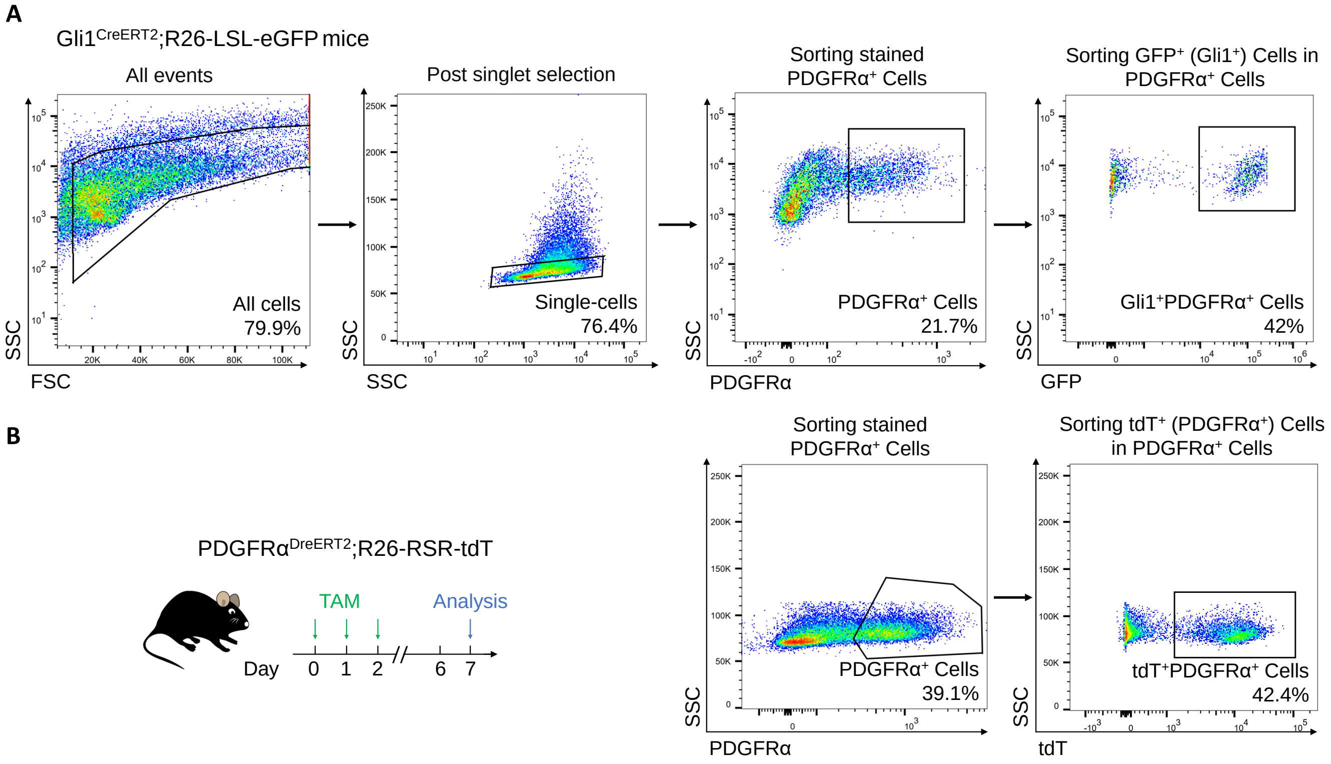

Supplementary Figure 2. FACS strategies for isolating Gli1$^+$PDGFRα$^+$ cells. (A) FACS analysis of the proportion of Gli1$^+$PDGFRα$^+$ cells in Gli1^{CreERT2};R26-LSL-eGFP mice. **(B)** FACS analysis of the labeling efficiency of PDGFRα^{DreERT2};R26-RSR-tdT mice.

Supplementary Figure 3

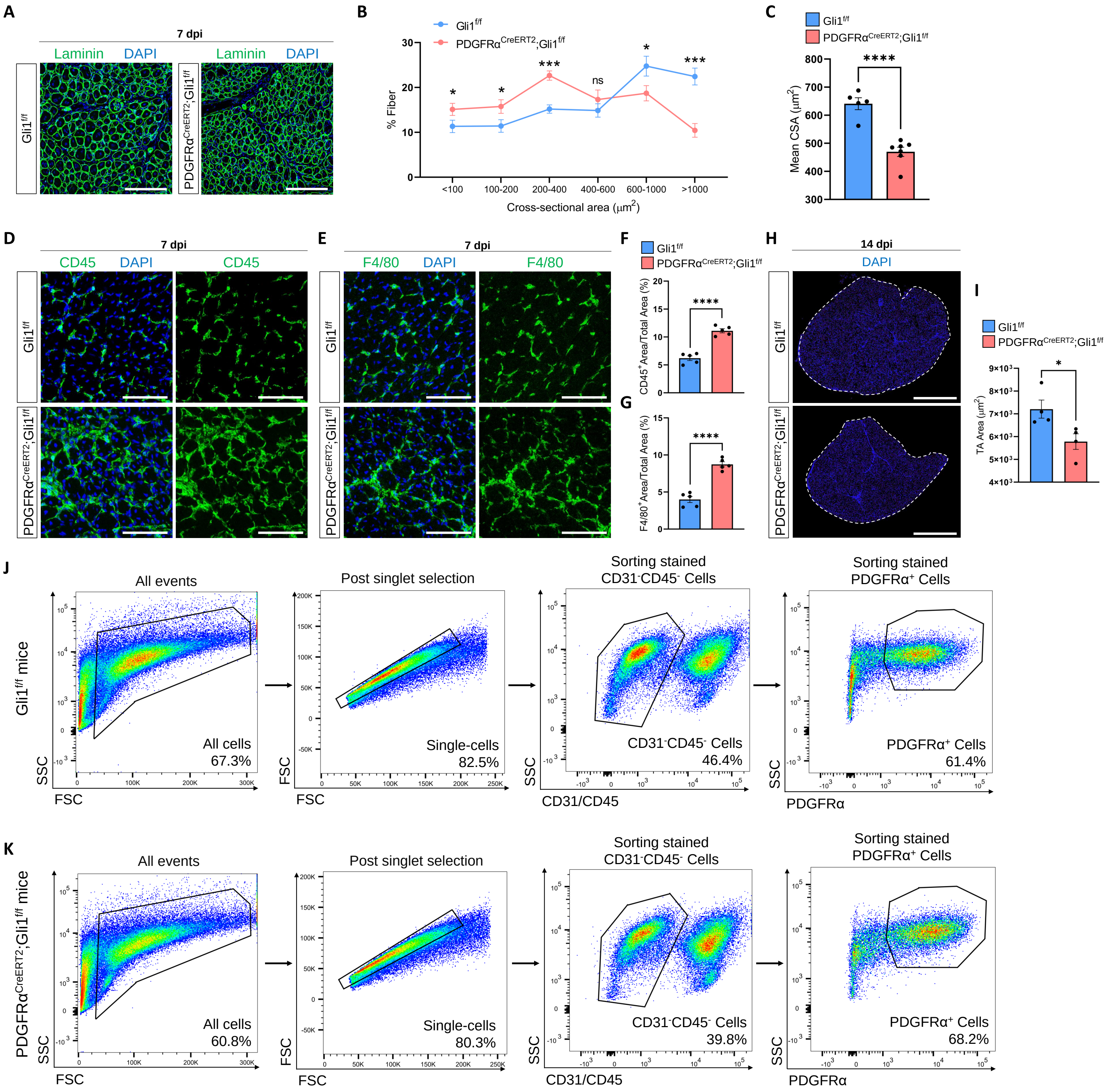

Supplementary Figure 3. FACS strategies for isolating FAPs at 7 dpi following CTX-induced injury. **(A)** IF staining of laminin (green) and DAPI (blue) of TA muscle sections from PDGFRα^{CreERT2};Gli1$^{f/f}$ mice at 7 dpi following CTX-induced injury. Scale bar, 200 μm. **(B)** Quantification of the myofiber CSA distribution in CTX-injured TA muscles (n = 5-7). Data are presented as mean ± SEM, $^{*}P < 0.05$, $^{***}P < 0.001$, ns indicates not significant. **(C)** Quantification of the mean CSA in CTX-injured TA muscles (n = 5-7). Data are presented as mean ± SEM, $^{****}P <$ 0.0001. **(D)** IF staining of CD45 (green) and DAPI (blue) of TA muscle sections from PDGFRα^{CreERT2};Gli1$^{f/f}$ mice at 7 dpi following CTX-induced injury. Scale bar, 100 μm. **(E)** IF staining of F4/80 (green) and DAPI (blue) of TA muscle sections from PDGFRα^{CreERT2};Gli1$^{f/f}$ mice at 7 dpi following CTX-induced injury. Scale bar, 100 μm. **(F)** Quantification of the CD45^{+} area within the total area (n = 5). Data are presented as mean ± SEM, $^{****}P < 0.0001$. **(G)** Quantification of the F4/80^{+} area within the total area in CTX-injured TA muscles (n = 5). Data are presented as mean ± SEM, $^{****}P < 0.0001$. **(H)** IF staining of DAPI (blue) of TA muscle sections from PDGFRα^{CreERT2};Gli1$^{f/f}$ mice at 14 dpi following CTX-induced injury. Scale bar, 1 mm. **(I)** Quantification of the TA area (n = 4). Data are presented as mean ± SEM, $^{*}P < 0.05$. **(J)** The FACS strategy to isolating FAPs from Gli1$^{f/f}$ mice on day 7 following CTX-induced injury. **(K)** The FACS strategy to isolating FAPs from PDGFRα^{CreERT2};Gli1$^{f/f}$ mice on day 7 following CTX-induced injury. All numbers (n) are biologically independent experiments.

Supplementary Figure 4

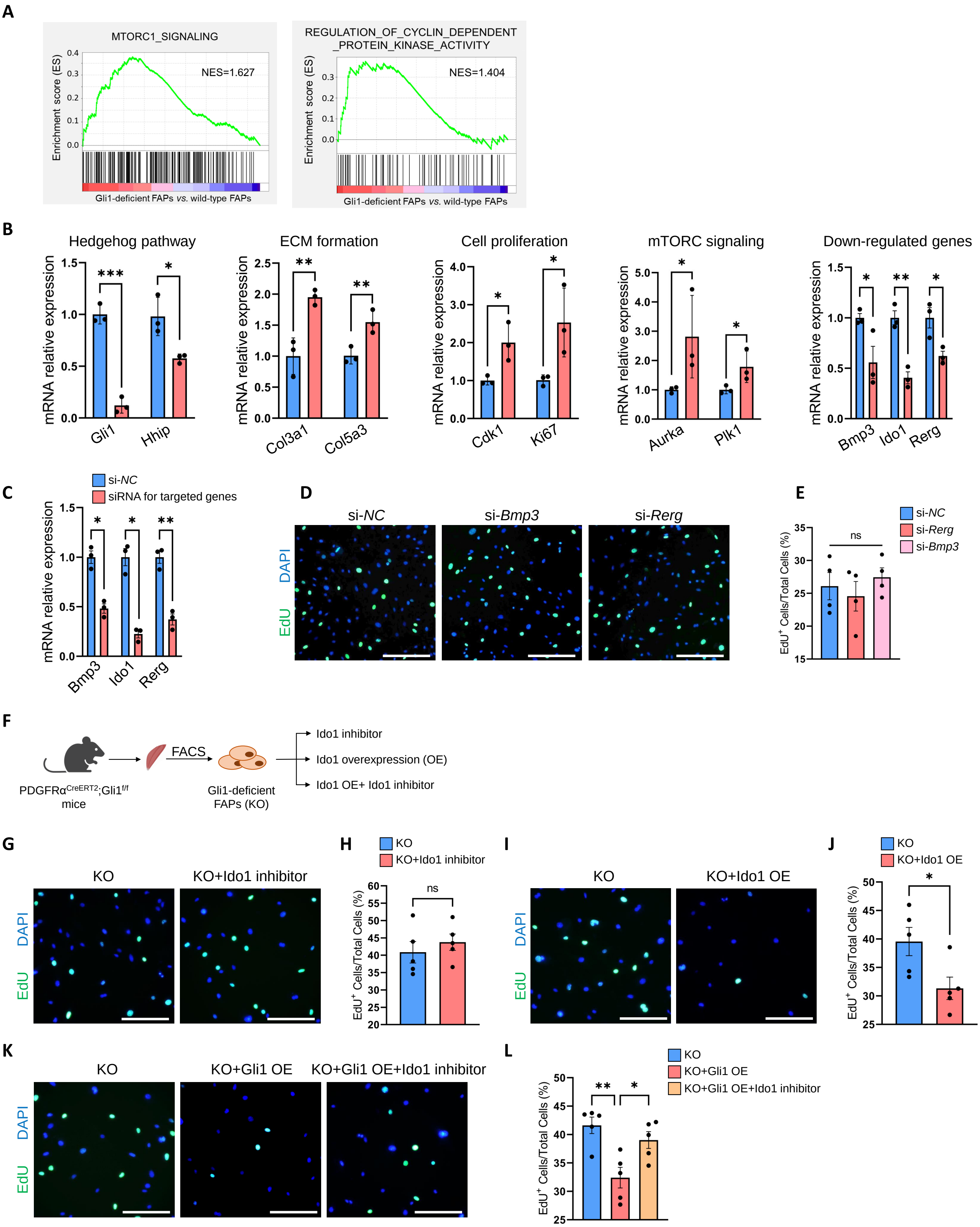

Supplementary Figure 4. Silencing of *Bmp3* and *Rerg* does not affect FAP proliferation. (A) Gene set enrichment analysis (GSEA) highlighting upregulated mTORC1 and regulation of cyclin dependent protein kinase activity. **(B)** The validation of DEGs by RT-qPCR (n = 3). Data are presented as mean ± SEM, $^{*}P < 0.05$, $^{**}P < 0.01$, $^{***}P < 0.001$. **(C)** Knock-down efficiency of si-*Bmp3*, si-*Ido1*, and si-*Rerg*. Data are presented as mean ± SEM, $^{*}P < 0.05$, $^{**}P < 0.01$. **(D)** EdU staining of FAPs treated with si-*Bmp3* and si-*Rerg*. Scale bar, 200 μm. **(E)** Quantification of the proportion of EdU^{+} cells following si-*Bmp3* and si-*Rerg* treatment (n = 4). Data are presented as mean ± SEM, ns indicates not significant. **(F)** Scheme of the experimental strategy for Ido1 and Gli1 modulation. **(G)** EdU staining of Gli1-deficient FAPs treated with or without Ido1 inhibitor. Scale bar, 200 μm. **(H)** Quantification of the proportion of EdU^{+} cells with or without Ido1 inhibitor treatment (n = 5). Data are presented as mean ± SEM, ns indicates not significant. **(I)** EdU staining of Gli1-deficient FAPs treated with or without Ido1 overexpression. Scale bar, 200 μm. **(J)** Quantification of the proportion of EdU^{+} cells with or without Ido1 overexpression (n = 5). Data are presented as mean ± SEM, $^{*}P < 0.05$. **(K)** EdU staining of Gli1-deficient FAPs with or without Gli1 overexpression and subsequent Ido1 inhibitor treatment. Scale bar, 200 μm. **(L)** Quantification of the proportion of EdU^{+} cells with or without Gli1 overexpression and subsequent Ido1 inhibitor treatment (n = 5). Data are presented as mean ± SEM, $^{*}P < 0.05$, $^{**}P < 0.01$. All numbers (n) are biologically independent experiments.

Supplementary Figure 5

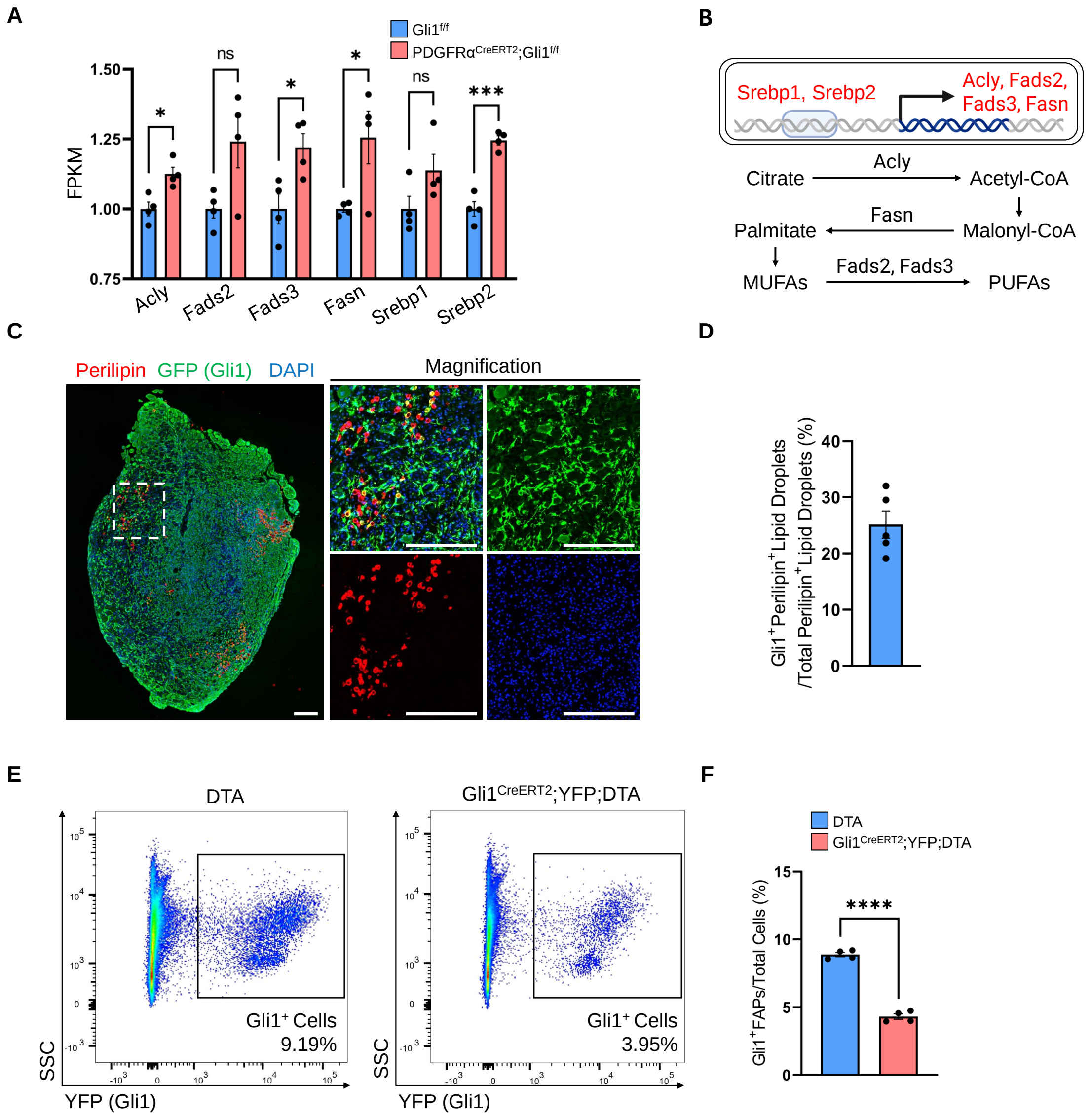

Supplementary Figure 5. Gli1-deficient FAPs exhibit enhanced fatty acid metabolism and increased adipogenic potential. (A) FPKM of key genes involved in fatty acid synthesis (n = 4). Data are presented as mean ± SEM, $^{*}P < 0.05$, $^{***}P < 0.001$, ns indicates not significant. **(B)** Scheme of the key genes involved in fatty acid synthesis. MUFAs, monounsaturated fatty acids; PUFAs, polyunsaturated fatty acids. **(C)** IF staining for perilipin (red), GFP (green), and DAPI (blue) in TA muscle sections from $Gli1^{CreERT2}$;R26-LSL-eGFP mice and control mice following glycerol-induced injury. Scale bar, 250 μm. **(D)** Quantification of the proportion of $Gli1^{+}perilipin^{+}$ lipid droplets within total $perilipin^{+}$ lipid droplets (n = 5). Data are presented as mean ± SEM. **(E)** FACS analysis of $Gli1^{+}$ cells within the total cells in $Gli1^{CreERT2}$;YFP;DTA mice. **(F)** Quantification of the DTA efficiency (n = 4). Data are presented as mean ± SEM, $^{****}P < 0.0001$. All numbers (n) are biologically independent experiments.

Supplementary Figure 6

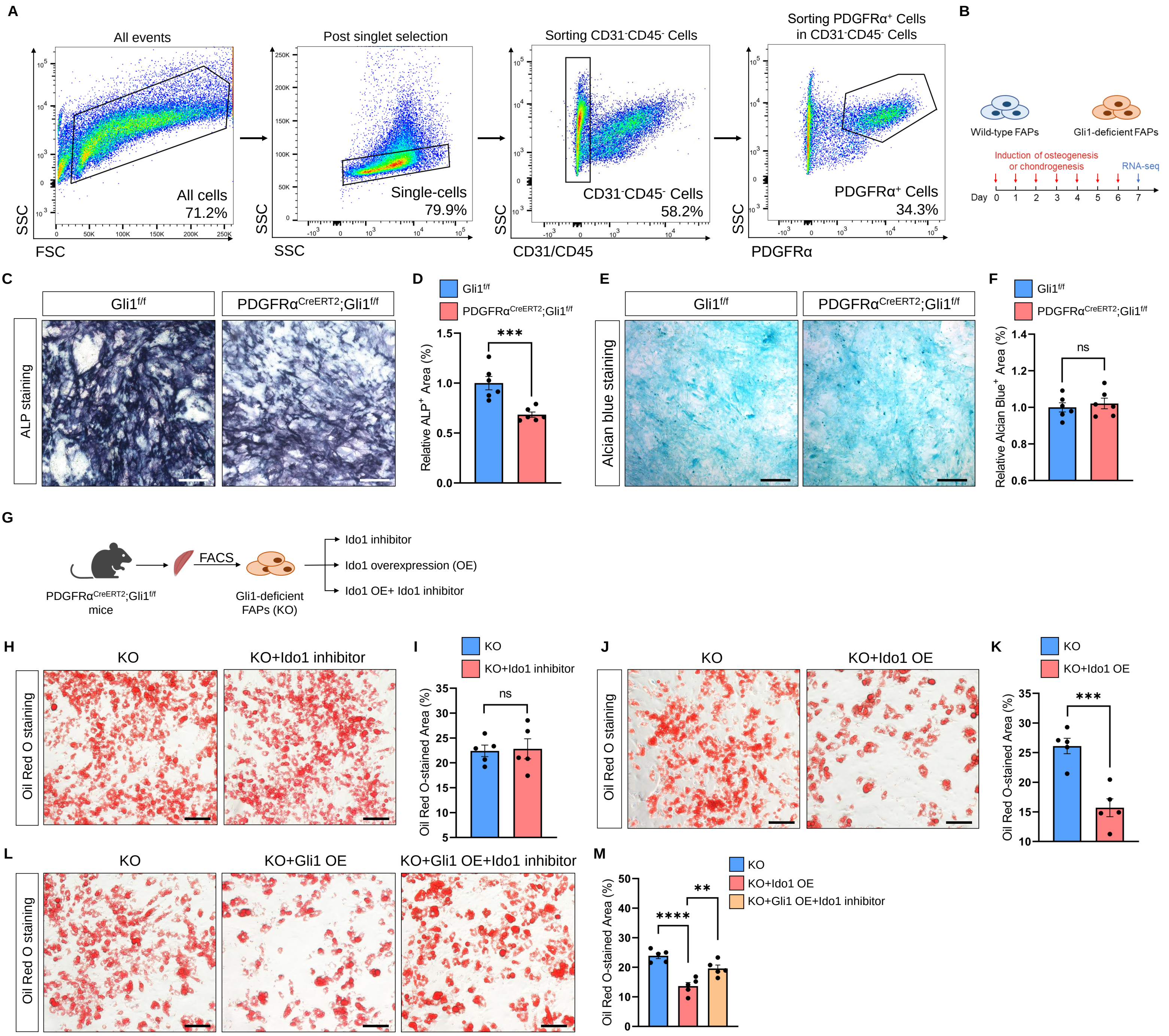

Supplementary Figure 6. Loss of Gli1 in FAPs significantly impairs osteogenic capacity without affecting chondrogenic potential. **(A)** Sorting strategy for isolating FAPs from PDGFRα^{CreERT2};Gli1$^{f/f}$ mice using FACS. **(B)** Scheme of the experiment of osteogenic differentiation and chondrogenic differentiation of FAPs. **(C)** ALP staining of osteogenic differentiation in FAPs from PDGFRα^{CreERT2};Gli1$^{f/f}$ mice. Scale bar, 200 μm. **(D)** Quantification of the relative ALP^{+} area. Data are presented as mean ± SEM, $^{***}P < 0.001$. **(E)** Alcian blue staining of chondrogenic differentiation in FAPs from PDGFRα^{CreERT2};Gli1$^{f/f}$ mice. Scale bar, 200 μm. **(F)** Quantification of the relative Alcian blue^{+} area. **(G)** Scheme of the experimental strategy for Ido1 and Gli1 modulation. **(H)** Oil red O staining of Gli1-deficient FAPs treated with or without Ido1 inhibitor. Scale bar, 200 μm. **(I)** Quantification of the proportion of Oil red O-stained area with or without Ido1 inhibitor treatment (n = 5). Data are presented as mean ± SEM, ns indicates not significant. **(J)** Oil red O staining of Gli1-deficient FAPs treated with or without Ido1 overexpression. Scale bar, 200 μm. **(K)** Quantification of the proportion of Oil red O-stained area with or without Ido1 overexpression (n = 5). Data are presented as mean ± SEM, $^{***}P < 0.001$. **(L)** Oil red O staining of Gli1-deficient FAPs with or without Gli1 overexpression and subsequent Ido1 inhibitor treatment. Scale bar, 200 μm. **(M)** Quantification of the proportion of Oil red O-stained area with or without Gli1 overexpression and subsequent Ido1 inhibitor treatment. Data are presented as mean ± SEM, $^{**}P < 0.01$, $^{****}P < 0.0001$. All numbers (n) are biologically independent experiments.

Supplementary Table 1. List of primary antibodies used for experiments

Primary antibody	Species	Dilution	Applications	Source	Catalog number
Laminin	Rabbit	1:300	IF	Abcam	ab11575
Laminin	Rat	1:300	IF	Merck	L0663
PDGFRα	Goat	1:100	IF	R&D	AF1062
eMyHC	Mouse	1:20	IF	DSHB	AB_528358
Perilipin	Rabbit	1:500	IF	CST	9349S
mcherry	Goat	1:500	IF	SICGEN	AB0081-200
GFP	Chicken	1:500	IF	Abcam	Ab13970
Gli1	Rabbit	1:1000	WB	CST	2534S
GAPDH	Rabbit	1:1000	WB	CST	2118S
CD31 (FITC-conjugated)	Rat	1:100	FACS	BioLegend	157214
CD45 (FITC-conjugated)	Rat	1:100	FACS	BioLegend	160212
Sca1 (APC-conjugated)	Rat	1:100	FACS	BioLegend	108112
PDGFRα (APC-conjugated)	Rat	1:100	FACS	Invitrogen	17-1401-81

Supplementary Table 2. List of pimers used for experiments

Genes	Pimer sequences (5'-3')
Gli1	Fw – CCAAGCCAACTTTATGTCAGGG<br>Rv – AGCCCGCTTCTTTGTTAATTTGA
Hhip	Fw –TGAAGATGCTCTCGTTTAAGCTG<br>Rv – CCACCACACAGGATCTCTCC
Col3a1	Fw –CCTGGCTCAAATGGCTCAC<br>Rv – CAGGACTGCCGTTATTCCCG
Col5a3	Fw – CGGGGTACTCCTGGTCCTAC<br>Rv – GCATCCCTACTTCCCCCTTG
Cdk1	Fw – AGAAGGTACTTACGGTGTGGT<br>Rv – GAGAGATTTCCCGAATTGCAGT
Ki67	Fw – ATCATTGACCGCTCCTTTAGGT<br>Rv – GCTCGCCTTGATGGTTCCT
Aurka	Fw – CTGGATGCTGCAAACGGATAG<br>Rv – CGAAGGGAACAGTGGTCTTAACA
Plk1	Fw – CTTCGCCAAATGCTTCGAGAT<br>Rv – TAGGCTGCGGTGAATTGAGAT
Bmp3	Fw – AACGATGCTGCCATTTCT<br>Rv – CTTCCTCCTCTCAACCGA
Ido1	Fw – CAAAGCAATCCCCACTGTATCC<br>Rv – ACAAAGTCACGCATCCTCTTAAA
Rerg	Fw – GAGAAGGCTTCGTGTTGGTTTACG<br>Rv – TTGTTTCCGACCAAGATGAGAGTCAC
Scd4	Fw – GCCCACTTGCCACAAGAGAT<br>Rv – GTAGCTGGGGTCATACAGATCA
Cbr1	Fw – TCAATGACGACACCCCCTTC<br>Rv – CCTCTGTGATGGTCTCGCTTC
Fgl1	Fw – CCCTGTCAGGAACTTTTCATCC<br>Rv – CGGTAGTAAACACCGTTCAGGT
Lrpap1	Fw – CACAACCTCAACGTCATCCTG<br>Rv – AGCACATTGTACTCCTGGATCTT
Notch3	Fw – TGCCAGAGTTCAGTGGTGG<br>Rv – CACAGGCAAATCGGCCATC
Heyl	Fw – CAGCCCTTCGCAGATGCAA<br>Rv – CCAATCGTCGCAATTCAGAAAG
Lfng	Fw – CGAGGTGCATAGCCTCTCC<br>Rv – GCGAGGGGACAGAACTTCG
Gapdh	Fw – AGGTCGGTGTGAACGGATTTG<br>Rv – TGTAGACCATGTAGTTGAGGTCA
